# Light control of droplets on photo-induced charged surfaces

**DOI:** 10.1093/nsr/nwac164

**Published:** 2022-08-17

**Authors:** Fang Wang, Meijin Liu, Cong Liu, Chao Huang, Lidong Zhang, Anyang Cui, Zhigao Hu, Xuemin Du

**Affiliations:** Institute of Biomedical and Health Engineering, Shenzhen Institute of Advanced Technology, Chinese Academy of Sciences, Shenzhen 518055, China; Institute of Biomedical and Health Engineering, Shenzhen Institute of Advanced Technology, Chinese Academy of Sciences, Shenzhen 518055, China; Institute of Biomedical and Health Engineering, Shenzhen Institute of Advanced Technology, Chinese Academy of Sciences, Shenzhen 518055, China; University of Chinese Academy of Sciences, Beijing 100049, China; Institute of Biomedical and Health Engineering, Shenzhen Institute of Advanced Technology, Chinese Academy of Sciences, Shenzhen 518055, China; Department of Chemistry and Molecular Engineering, East China Normal University, Shanghai 200241, China; Technical Center for Multifunctional Magneto-Optical Spectroscopy (Shanghai), Engineering Research Center of Nanophotonics and Advanced Instrument (Ministry of Education), Department of Physics, School of Physics and Electronic Science, East China Normal University, Shanghai 200241, China; Technical Center for Multifunctional Magneto-Optical Spectroscopy (Shanghai), Engineering Research Center of Nanophotonics and Advanced Instrument (Ministry of Education), Department of Physics, School of Physics and Electronic Science, East China Normal University, Shanghai 200241, China; Institute of Biomedical and Health Engineering, Shenzhen Institute of Advanced Technology, Chinese Academy of Sciences, Shenzhen 518055, China

**Keywords:** light control, droplet manipulation, smart surface, liquid metal, ferroelectric

## Abstract

The manipulation of droplets plays a vital role in fundamental research and practical applications, from chemical reactions to bioanalysis. As an intriguing and active format, light control of droplets, typically induced by photochemistry, photomechanics, light-induced Marangoni effects or light-induced electric fields, enables remote and contactless control with remarkable spatial and temporal accuracy. However, current light control of droplets suffers from poor performance and limited reliability. Here we develop a new superamphiphobic material that integrates the dual merits of light and electric field by rationally preparing liquid metal particles/poly(vinylidene fluoride-trifluoroethylene) polymer composites with photo-induced charge generation capability in real time, enabling light control of droplets on the basis of photo-induced dielectrophoretic force. We demonstrate that this photo-induced charged surface (PICS) imparts a new paradigm for controllable droplet motion, including high average velocity (∼35.9 mm s^−1^), unlimited distance, multimode motions (e.g. forward, backward and rotation) and single-to-multiple droplet manipulation, which are otherwise unachievable in conventional strategies. We further extend light control of droplets to robotic and bio-applications, including transporting a solid cargo in a closed tube, crossing a tiny tunnel, avoiding obstacles, sensing the changing environment via naked-eye color shift, preparing hydrogel beads, transporting living cells and reliable biosensing. Our PICS not only provides insight into the development of new smart interface materials and microfluidics, but also brings new possibilities for chemical and biomedical applications.

## Teaser

Photo-induced charged superamphiphobic surfaces enable flexible and reliable droplet manipulation for droplet robots and bio-applications.

## INTRODUCTION

Droplet manipulation is crucial for both scientific research and practical applications, such as chemical reactions, high-throughput biological analysis and point-of-care diagnostics [1–9]. Conversion of external physical fields to droplet motion is an attractive and active format [3–7]. Despite remarkable progress, droplet manipulation with these physical fields, such as electric fields and magnetic fields, is limited by poor flexibility and short longevity owing to the requirement of large equipment, sophisticated electrode design and additional electric/magnetic responsive agents [8,9]. As one of the most intriguing physical fields with regard to manipulating droplets, light can overcome the above drawbacks via various strategies. Typically, the conversion of light energy to droplet motions involves using driving forces on droplets to overcome the interfacial resistance forces, in which the driving forces can be induced by photochemistry (photoresponsive agents), [10,11] photomechanics (light-induced capillary forces), [12] light-induced Marangoni effects, [13] or light-induced electric fields [4,5,14–16]. However, a large part of these driving forces is offset by the interfacial resistance forces, leading to the poor performance of droplet manipulations, including relatively low velocity, short distance and a lack of flexibility [8,9,17]. Unfortunately, the photoresponsive surfactants, UV light or the caused optical damage makes it difficult to achieve biocompatible and reliable manipulation, thus making it challenging to apply the method to chemical and biological domains where well-controlled manipulation of droplets is preferred [8,9,17–21].

**Figure 1.**
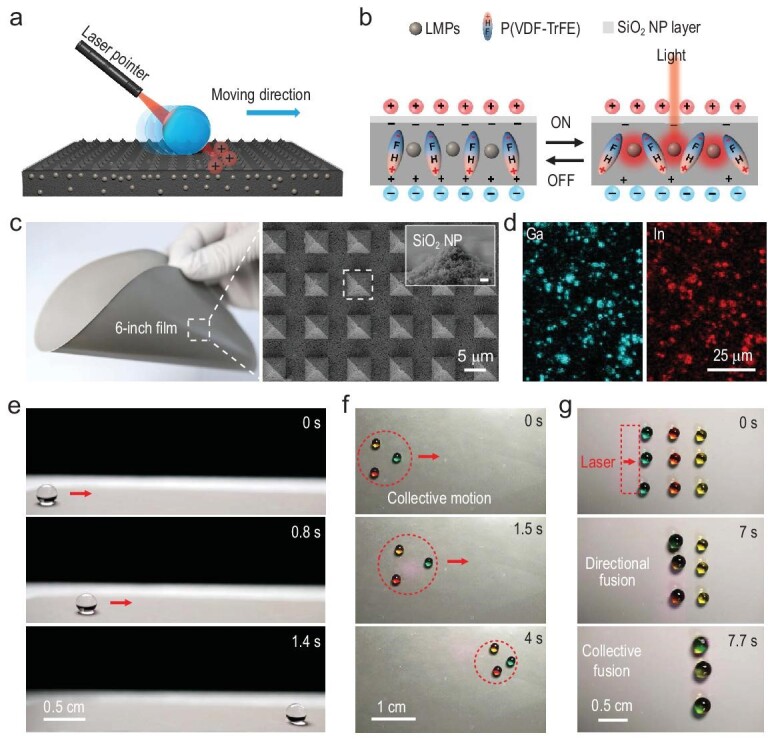
PICS fabrication. (a and b) Schematic illustrations of light control of a droplet on the PICS with real-time and *in-situ* generation of free surface charges. (c) Photograph of 5% LMP/P(VDF-TrFE) film and its surface morphologies. The scanning electron microscopy (SEM) images show the micro-pyramidal array and the sprayed fluorinated SiO_2_ NP coatings (scale bar: 500 nm). (d) EDS images of Ga-In LMPs in the PICS film. (e) Side-view photographs of an 8-μL water droplet motion with an average velocity of 35.9 mm s^−1^ on a PICS upon exposure to 808-nm NIR irradiation (irradiation angle, 45°; average power density, 996 mW cm^−2^; Movie S1). (f) Top-view photographs of the collective motions of three dyed water droplets (4 μL) on a PICS upon exposure to a single laser beam (average power density, 996 mW cm^−2^; Movie S2). (g) Photographs of the collectively directional fusion of three dyed water droplets (4 μL) in a step-by-step manner on a PICS under the control of a single handheld laser pointer.

Here we develop a new superamphiphobic material that integrates the dual merits of light and electric field by rationally preparing liquid metal particles/polyvinylidene fluoride trifluoroethylene (LMPs/P(VDF-TrFE)) polymer composites with high efficiency and durable photo-induced charge regeneration capability, enabling distinctive light control of droplets on the photo-induced charged surface (PICS). Such a PICS contains three core components (Fig. 1a–d): (i) micro-size LMPs owing to its superior photothermal and thermally conductive properties; (ii) P(VDF-TrFE) copolymer for its excellent ferroelectric and mechanical behaviors [22–24]; (iii) micro-pyramidal structures and low-surface-energy coatings of fluorinated silica nanoparticles (SiO_2_ NPs) for enhancing the superamphiphobicity. Leveraging the synergistic effect of these components, a PICS possesses the superior capability of real-time and *in-situ* photo-induced charge generation upon exposure to near-infrared (NIR, 808 nm) light irradiation. Such a PICS introduces a new paradigm for light control of droplets, characterized by high average velocity (∼35.9 mm s^−1^), single-to-multiple droplets and collectively directional fusion guided by a single laser beam. Additionally, there is no need for any large equipment, electrodes or additives (Fig. 1e–g, Movies S1 and S2 in the online supplementary materials).

## RESULTS AND DISCUSSION

### Fabrication of photo-induced charged surfaces

We firstly filled a prefabricated silicon mold with a pre-prepared composite solution of LMPs and P(VDF-TrFE), which was followed by complete solidification (see Experimental section in Supplementary Data). After peeling the solidified film from the mold, an electric field was applied to induce dipoles orientation within the LMPs/P(VDF-TrFE), and fluorinated SiO_2_ NPs were finally sprayed to form micro-nano hierarchical structures for increasing the surface roughness and lowering the surface energy (Figs S1 and S2). The PICS film (∼112 μm in thickness) can be made as large as 6 inches, and exhibits outstanding flexibility and excellent integrity (Fig. 1c, Fig. S3a). We noted that the pre-synthesized Ga-In LMPs were well dispersed in the composite film, constructing an effective interface between the LMPs and the P(VDF-TrFE) copolymer, as evidenced by X-ray energy-dispersive spectroscopy (EDS) and atomic force microscopy (AFM) images (Fig. 1d, Fig. S3b). This integrated interface is attributed to the extraordinary liquid–solid interface between the LMPs and the P(VDF-TrFE) copolymers, and the strong electrostatic interactions between the positive-charged LMPs and the negative-charged fluorine atoms. Such a PICS shows excellent superamphiphobicity owing to the synergism between the increased surface roughness and lowered surface energy. Diverse liquid droplets, including water, concentrated sodium chloride solutions (0.1 M–1 M), aqueous glycerol solutions (20 v/v%–80 v/v%), ethylene glycol and 1, 4-butanediol, have a large contact angle (∼150°) and a very low roll-off angle (<1°) on the PICS (Figs S4 and S5).

### Photo-induced charge generation of a PICS

**Figure 2.**
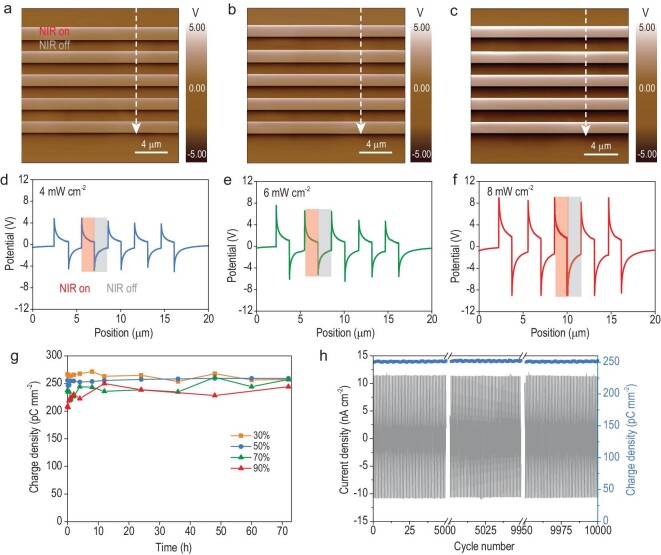
Charge generation properties of a PICS. (a–c) The SKPM images of a PICS as periodically irradiated by 808-nm NIR light with different power densities of 4 mW cm^−2^, 6 mW cm^−2^ and 8 mW cm^−2^, respectively. (d–f) The surface potential changes along the scanning line on the SKPM images of the PICS as periodically irradiated by 808-nm NIR with different power densities of 4 mW cm^−2^, 6 mW cm^−2^ and 8 mW cm^−2^, respectively, showing that the surface potential can be tuned by facilely adjusting the power density of NIR light irradiation. (g) The stability of surface charge density under different relative humidity conditions (30%, 50%, 70% and 90%). (h) The durability of photothermal pyroelectric current density and charge density of a PICS after 10 000 cycles of periodical irradiation.

Upon exposure to NIR light irradiation, the embedded LMPs in the PICS rapidly absorb and convert NIR light into heat, thus raising the temperature at the irradiated area (Fig. 1b). The localized temperature rise induces a decrease in P(VDF-TrFE) polarization due to the orientation loss of the dipoles within the copolymer, thus generating free surface positive charges at the irradiated spot as compensation. After turning off the NIR irradiation, the local temperature decreases, and thus the LMP/P(VDF-TrFE) polarization increases as the dipoles regain their initial orientations, leading to the final disappearance of free positive surface charges. This distinctive charge generation capability of the PICS is clearly revealed by scanning Kelvin probe microscopy (SKPM), which shows the real-time and *in-situ* generation/disappearance of the free surface charges upon exposure to ON/OFF NIR irradiation (Fig. 2a, Fig. S6). Notably, the surface charge density, and hence the surface potential, increases as the laser power density is increased, as shown in Fig. 2a–f.

To quantitively investigate the charge generation property, the PICS films were further sandwiched with deposited indium-tin-oxide (ITO) layers on both sides, as electrodes. Upon exposure to NIR light irradiation, the packed PICS can create a photothermal pyroelectric current (*I*) under short-circuit conditions (Fig. S7). For a PICS with an irradiated area of *S*, the *I* is expressed as [25]

(1) \begin{equation*} I = pS\frac{{dT}}{{dt}}, \end{equation*}

where *p* is the pyroelectric coefficient, *S* is the irradiated area and ($\frac{{dT}}{{dt}}$) is the temperature change rate, respectively. Thus, the photo-induced surface charge density (*σ*) on the PICS can be depicted as follows:

(2) \begin{equation*} \sigma = p\Delta T, \end{equation*}

where Δ*T* is the temperature change. This relationship shows that *σ* is proportional to Δ*T*. Thus, the photo-induced surface charge density can be enhanced by increasing the mass ratio of the LMPs or the power density of NIR irradiation, which can lead to a larger Δ*T* (Figs S8–S10, Discussion 2.1, Supplementary Data). Typically, the 5% LMP/P(VDF-TrFE) film with the highest photothermal conversion efficiency (*η*) developed the largest surface charge density of 253 pC mm^−2^, which is five times higher than that of the pristine P(VDF-TrFE) sample (50 pC mm^−2^, Fig. S10a). Remarkably, such charge generation capability of the PICS is superior to the P(VDF-TrFE) films embedded with other photothermal agents, including gold nanorods, carbon nanotubes and graphene oxide nanosheets, for two primary reasons: first, the excellent interfaces between the LMPs and P(VDF-TrFE) copolymers; second, the overwhelmingly photothermal and thermal conductive properties of LMPs (Fig. S11) [26,27]. More surprisingly, this unique charge generation capability of the PICS exhibits no apparent degradation even in extreme environments, including high relative humidity (∼90%) for 72 hours and high temperature (70°C), which has been impossible in previous work (Fig. 2g, Fig. S12) [28,29]. Notably, the output current density and charge density of our PICS remain at the stable high levels of 21.9 nA cm^−2^ and 252 pC mm^−2^ (peak to peak) even after 10 000 ON/OFF irradiation cycles (Fig. 2h). Such results indicate the outstanding efficiency, superior durability and stability of the photo-induced charge regeneration in the PICS, which is critical for light control of droplets.

### Mechanism and performance of light control of droplets

**Figure 3.**
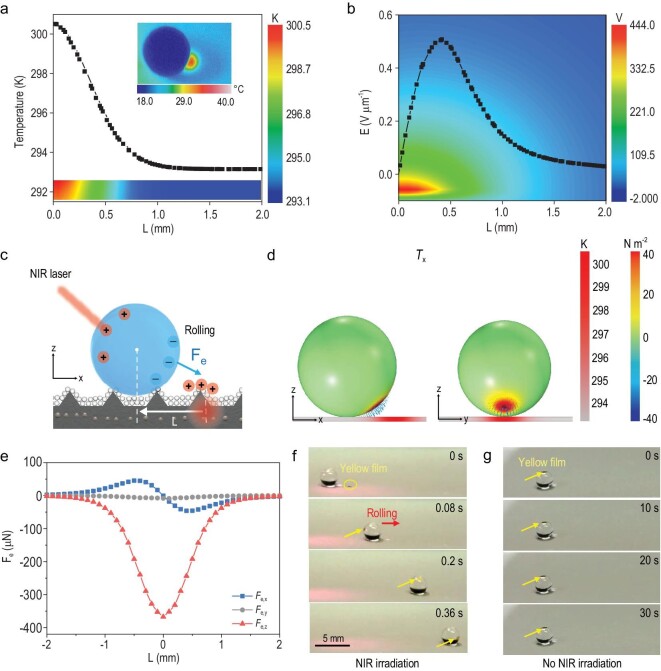
Mechanism of a PICS for droplet dynamics. (a) Temperature mapping in the PICS is simulated using COMSOL. The inset is the infrared thermal imaging picture of an 8-μL water droplet on a PICS upon exposure to NIR irradiation for 0.2 s (irradiation angle, 45°; power density, 996 mW cm^−2^). (b) Electrical field mapping in the PICS, showing the peak near the edge of the irradiated spot (radius: 0.4 mm). (c) Schematic illustration of the mechanism of the PICS for droplet rolling on the basis of the photo-induced dielectrophoretic force (*F*_e_, driving force). *L* is the x-direction distance from the irradiated spot center to the droplet center. (d) Maxwell stress tensor (left, x–z plane view; right, y–z plane view) in the x-direction (*T*_x_) exerting on the droplet (8 μL) upon exposure to NIR irradiation (25 mW) is obtained by simulation. The directions and magnitudes of dielectrophoretic surface stresses are represented by the directions and lengths of blue arrows, respectively. (e) The *F*_e, x_, *F*_e, y_ and *F*_e, z_ can be adjusted by varying *L*. (f) Photographs of a yellow plastic piece suspended in an 8-μL water droplet marking the rolling behavior of the droplet on the PICS upon exposure to the NIR irradiation (Movie S3). (g) Photographs of a motionless yellow plastic piece suspended in an 8-μL water droplet on the PICS without NIR irradiation as a control.

We next performed a numerical model to reveal the underlying mechanism for light control of droplets. As shown in Fig. 3a, NIR light passing through a droplet forms an irradiated spot on the PICS. The light-induced temperature increase at the irradiated spot results in the real-time generation of free charges and thus an electric field (Fig. 3b). The light-induced electric field induces a dielectrophoretic force (*F*_e_) that exerts on the droplet. This can be expressed as (Fig. 3c, Discussion 2.2, Supplementary Data) [6,30]

(3) \begin{equation*} {{{\bf F}}}_e \,{=}\, \oint {{\bf{T}} \cdot {\bf{n}}dS} ,{T}_{ij} \,{=}\, {\varepsilon }_0{\varepsilon }_r\!\left({E}_i{E}_j\,{-}\, \frac{1}{2}{\delta }_{ij}{E}^2\right), \end{equation*}

where **T** is the Maxwell stress tensor, **n** is the surface unit normal, *ϵ*_0_ and *ϵ*_r_ are the permittivity of vacuum and the relative permittivity of water, *δ*_ij_ is the Kronecker delta notation and *E* is the electric field intensity, respectively. Maxwell stress tensors in x direction (*T*_x_), y direction (*T*_y_) and z direction (*T*_z_) exerting on the droplet are obtained by simulation, as shown in Fig. 3d and Fig. S13. By integrating the Maxwell stress tensors, dielectrophoretic force components *F*_e, x_, *F*_e, y_ and *F*_e, z_ are calculated, respectively. Notably, the *F*_e, y_ perpendicular to the *L* direction (*L*, the x-direction distance from the irradiated spot center to the droplet center) is negligible owing to the symmetric *T*_y_ (Fig. 3e) [6]. By comparison, the *F*_e, x_ first increases to the maximum value near the edge of the irradiated spot and then decreases to zero at the irradiated spot center (*L* = 0) with decreasing *L*, while the *F*_e, z_ continuously increases with decreasing *L* and finally reaches the maximum value at the irradiated spot center (*L* = 0). In addition, the dielectrophoretic force can be increased by increasing the charge density (*σ*), which depends on the temperature change (Figs S14 and S15). It is worth noting that the viscous energy dissipation can be negligible owing to the excellent superamphiphobic PICS, and therefore, the combined *F*_e, x_ and *F*_e, z_ enable the rolling behavior of the droplet on PICS (Fig. 3f and g). Such results suggest that the droplet dynamic behaviors can be adjusted by changing *L* and *σ*, which can be facilely tuned by varying the NIR irradiation angle (*α*) and distance between the laser beam and the droplet surface (*D*, Fig. S16).

**Figure 4.**
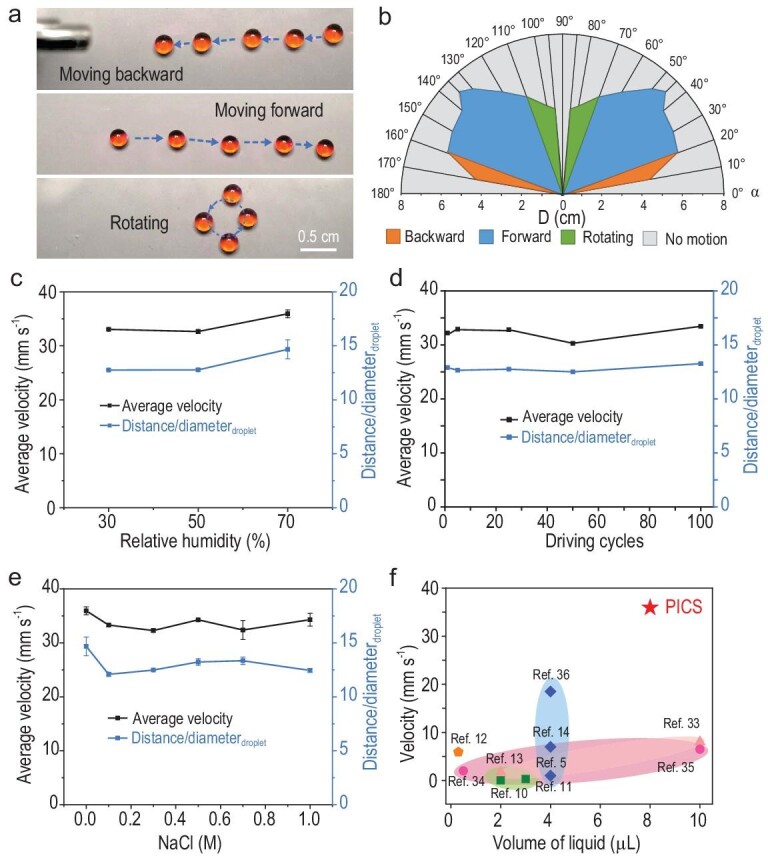
Multimode motion and performance control. (a) An 8-μL dyed droplet shows multimode motions, including moving forward (irradiation angle, 45°), backward (irradiation angle, 15°) and rotating (irradiation angle, 80°) via changing the irradiation angle of NIR light (Movie S4). (b) The phase diagram illustrates the droplet dynamic behaviors on the PICS via varying the NIR irradiation angle (*α*) and the distance between the laser beam and the water droplet surface (*D*). (c) The droplet motion on the PICS is not sensitive to relative humidity (30%, 50% and 70%). (d) Light control of a droplet on the PICS can last 100 cycles without obvious fatigue. (e) The universality of droplet motions for sodium chloride solutions with different concentrations (1 M NaCl droplet with an average velocity as high as 34.3 mm s^−1^). (f) Comparison of the droplet motion performances among our PICS with the reported photomechanics, photochemistry, light-induced Marangoni effect, heat and light-induced electric field strategies.

Our experiments further demonstrate that the droplet dynamic behaviors on the PICS are consistent with the simulation results. As shown in Fig. 4a and b, and Figs S17 and S18, the droplet can remain in backward motion, forward motion or rotation, or it can be stationary, by facilely changing *α* and *D* between the laser beam and the droplet surface (Movies S4–S6, Table S2). When 10° < *α* < 20° and 0 cm < *D* < 6 cm, the droplet shows backward motion. When 20° < *α* < 70° and 0 cm < *D* < 7.2 cm, the droplet shows forward motion. When 70° < *α* < 85° and 0 cm < *D* < 5.1 cm, the droplet rotates around the irradiated spot owing to the tangential dielectrophoretic force. When 85° < *α* < 90°, the droplet remains motionless. In contrast, the droplet keeps stationary when 0° ≤ *α* <10° or *D* beyond the range above, because the large *L* and low *σ* lead to a negligible dielectrophoretic force in the x–y plane. As 90° < *α* < 180°, the droplet performs similar motion modes to those discussed above; however, these on-demand multimode motions were impossible to achieve in previous strategies [29,31]. It is worth noting that the water droplet can move on the PICS upon exposure to NIR light irradiation, however, it cannot move on surfaces without charge generation capability or micro/nanostructures, respectively (Fig. S19). This further confirms that the droplet motion depends on the synergistic effect of both the outstanding photo-induced real-time charge generation and superamphiphobicity of the PICS. In addition, the negligible temperature gradient and volume change at/of the light-controlled droplet also indicate that the droplet motion is driven by the photo-induced dielectrophoretic force rather than the photothermal-induced Marangoni effect (Figs S20 and S21) [32,33].

Compared to the previously reported strategies, the distinctive advantages of the PICS allow us to manipulate droplets with superior performance. Specifically, an 8-μL water droplet can move at an average velocity of ∼35.9 mm s^−1^ (Movie S1), which is superior to those on conventional photoresponsive surfaces [8,9,17]. The motion velocity of the droplet can be adjusted by changing the moving velocity of the laser beam, LMP concentration and droplet volume, as shown in Figs S22–S25. In addition, the continuous motion distance of the water droplet can be as long as 100 mm, which is 40 times larger than its diameter (2.5 mm, Fig. S26, Movie S7). Since the droplet motions can be continuously driven by a moving laser, there is no theoretical limit to moving distance. It is worth noting that the droplet motion is not sensitive to humidity, and the outstanding performance of droplet motions on a PICS upon intermittent irradiation with 808-nm light can repeat for >100 cycles without obvious fatigue, benefitting from the superior capability of the photo-induced surface charge regeneration of the PICS (Fig. 4c and d). Furthermore, such robust manipulation is generic to diverse liquids, including concentrated sodium chloride solutions, aqueous glycerol solutions, ethylene glycol and 1,4-butanediol (Fig. 4e, Figs S27 and S28, Table S3, Movie S8). Compared to previous strategies, our PICS demonstrates superior overall performance with regard to the aspects of manipulating condition, droplet motion behaviors and reliability (Fig. 4f, Table S4) [5,10–14,33–36].

### Light control of droplets for robotic applications

**Figure 5.**
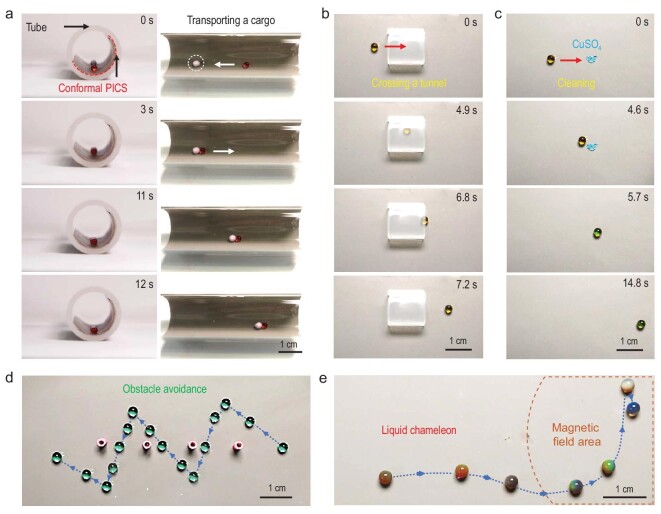
Droplet robots. (a) Side and front views of an 8-μL dyed water droplet for transporting a solid cargo in the inner tube with a conformed PICS film (808-nm NIR laser pointer). (b) A 2-μL dyed water droplet was guided by an 808-nm NIR laser pointer through a narrow tunnel. (c) A 2-μL dyed water droplet was guided by an 808-nm NIR laser pointer for the purpose of cleaning a powder sample. (d) The time-lapse trajectory of a 2-μL dyed water droplet for dynamic obstacle avoidance, navigated by an 808-nm NIR laser pointer. (e) Controllable motion of a 2-μL droplet robot (liquid chameleon) containing monodisperse Fe_2_O_3_@SiO_2_ ellipsoidal core-shell particles (5 wt%) driven by an 808-nm NIR laser pointer, which can change its color instantly when it senses the magnetic field change when a permanent magnet is placed beneath the film.

The flexible and precise light control of droplets on a PICS can be further harnessed for various robotic applications. First, an 8-μL water droplet can be facilely navigated by a handheld laser pointer in order to transport a solid cargo in a closed tube covered with a flexible PICS (Fig. 5a, Fig. S29, Movie S9) [37]. Second, the water droplet can be guided to pass through a very narrow tunnel (height <5 mm), work as a ‘cleaner’ for carrying a powder sample to a specific position, and even cross closely aligned obstacles (inter distance: ∼1 cm) with an on-demand pathway (Fig. 5b–d, Movies S10–S12), illustrating precise light control of droplet locomotion. Third, a droplet containing monodisperse ellipsoidal magnetic particles can serve as a ‘liquid chameleon’ enabling us to sense the varying environment via naked-eye color changes (Fig. 5e, Movie S13). The droplet robot with initial brown color is guided by a handheld laser pointer, and then changes its color to red owing to the rapid assembly of the disordered magnetic particles into periodical structures that diffract visible light (structure color) once the droplet robot moves closer to the magnetic field area. Moving into the magnetic field central area, the droplet further varies its color, instantly, from red to green and finally blue, due to the decrease in the interparticle distances in the droplet, which is induced by the increased magnetic field strength (Figs S30 and S31) [38–41].

**Figure 6.**
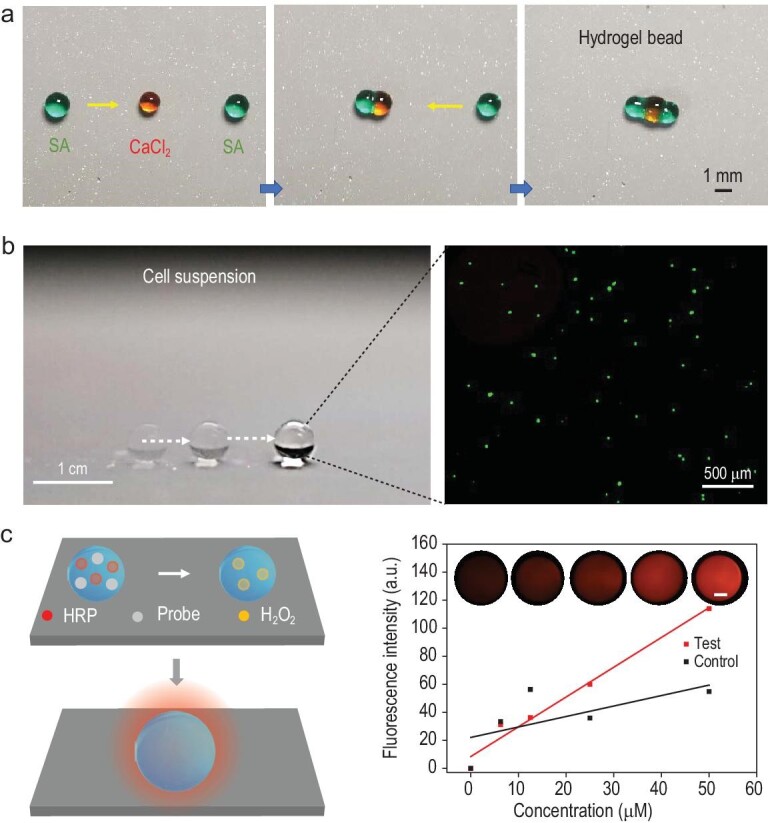
Bio-applications. (a) Controllable motions of SA (2 μL) and CaCl_2_ droplets (1 μL) for merging into snowman- and dumbbell-hydrogel beads, guided by an 808-nm NIR laser pointer (Movie S14). (b) Controllable motion of a 2-μL cell culture media droplet containing live cells, guided by an 808-nm NIR laser pointer. (c) Schematic illustration of light control of a droplet on the PICS for hydrogen peroxide (H_2_O_2_) detection (left); the fluorescence intensity of the test and control (right)—the inset is fluorescence images of the test (scale bar: 500 μm).

Light control of droplet robots on a PICS can be further extended to bio-applications. As shown in Fig. 6a, droplets containing high viscosity sodium alginate (SA, 2 wt%) and calcium chloride (CaCl_2_, 1 M) can even be guided to fuse into hydrogel beads with on-demand snowman and dumbbell morphologies (Fig. 6a). Surprisingly, a cell suspension droplet can also be transported at speed (∼32 mm s^−1^) and with high cell viability (∼98%), indicating the biocompatible manipulation condition (Fig. 6b, Fig. S32). Leveraging such robotic and biocompatible features, light control of droplets on a PICS can be further used for the detection of hydrogen peroxide (H_2_O_2_), which is a very key molecule in biology. Briefly, droplets (2 μL) containing horseradish peroxidase (HRP) and a fluorogenic probe were guided and then fused with the droplets containing H_2_O_2_ of different concentrations (0, 6.25, 12.5, 25 and 50 μM). As shown in Fig. 6c, the fluorescence intensities of the fused droplets followed a standard linear correlation (*R*: 0.99) to the H_2_O_2_ concentrations. In contrast, the control shows no obvious linear correlation between the fluorescence intensity and H_2_O_2_ concentration (*R*: 0.65) because the excessive temperature increase may affect the activity of the thermal-sensitive HRP and leads to unreliable detection (Fig. S33). In light of the above demonstrations, it is conceivable that our new strategy for light control of droplets could provide solutions for overcoming many existing limitations in chemical, biomedical and robotic fields where well-controlled manipulation of liquids is preferred [36].

## CONCLUSION

In summary, we develop a new superamphiphobic material that integrates the dual merits of light and electric field, and harness its high-efficiency and stable photo-induced charge regeneration capability for manipulating droplets. We demonstrate that the PICS provides a new paradigm for light control of droplets, which includes mild and biocompatible manipulating conditions, and highly flexible and precise droplet manipulation with various types, numbers and multimode motions. More advanced than the previous methods, our strategy not only endows light control of droplets with high average velocity, long distance and an on-demand pathway, but also produces unprecedented droplet robots and their reliable bio-applications, such as transporting a solid cargo, avoiding obstacles, sensing the changing environment and detecting biomolecules. We expect this new material with photo-induced charge regeneration capability to find uses in biology (e.g. sensing specific signals and stimulating living matter). We anticipate that our PICS will promote more advanced developments in smart interface materials and microfluidics, as well as their broad application in chemical and biomedical domains.

## Supplementary Material

nwac164_Supplemental_Files
