# Supplementary material for: Light control of droplets on photo-induced charged surfaces
Source: Natl Sci Rev. 2022 Aug 17;10(1):nwac164. doi: 10.1093/nsr/nwac164 (PMC9843135; doi:10.1093/nsr/nwac164)
Supplement: nwac164_Supplemental_Files [file nwac164_supplemental_files.zip › Supporting_Information.pdf]

Supporting Information

Light control of droplets on photo-induced charged surfaces

Fang Wang[#1], Meijin Liu[#1], Cong Liu[#1,2], Chao Huang[1], Lidong Zhang[3], Anyang Cui[4], Zhigao Hu[4], Xuemin Du[1]*

Dr. F. Wang, Dr. M. Liu, C. Liu, Dr. C. Huang, Prof. X. Du

[1]Institute of Biomedical & Health Engineering, Shenzhen Institute of Advanced Technology (SIAT), Chinese Academy of Sciences (CAS), Shenzhen, P. R. China.

[2]University of Chinese Academy of Sciences, Beijing, P. R. China.

[3]Department of Chemistry and Molecular Engineering, East China Normal University Shanghai, P. R. China.

[4] Technical Center for Multifunctional Magneto-Optical Spectroscopy (Shanghai), Engineering Research Center of Nanophotonics & Advanced Instrument (Ministry of Education), Department of Physics, School of Physics and Electronic Science, East China Normal University, Shanghai, P. R. China.

Correspondence to: *E-mail: xm.du@siat.ac.cn.

1. Experimental Section

1.1 Materials

Poly(vinylidene fluoride-trifluoroethylene) (P(VDF-TrFE), 70/30, M_w: 520,000-860,000) was purchased from Piezotech (Pierre-Benite, France). Sodium chloride (NaCl), calcium chloride ($CaCl_2$), copper sulfate ($CuSO_4 \cdot 5H_2O$), sodium alginate (SA), glycerol, dimethyl sulfoxide (DMSO), hydroquinone, chloroauric acid, silver nitrate,

sodium borohydride, tetraethyl orthosilicate (TEOS, 98%), 1*H*, 1*H*, 2*H*, 2*H*-perfluorodecyl trichlorosilane (PFDTS, 96%), ethylene glycol (EG), and 1, 4-butanediol (BD) were purchased from Aladdin Reagent Co., Ltd (Shanghai, China). Liquid metal eutectic gallium-indium (LM, 75.5% gallium and 24.5% indium) was purchased from Fanyada Electronic Technology Co., Ltd (Zhenjiang, China). Methyl cellulose was supplied by Alfa Aesar Chemical Reagent Co., Ltd (Tianjin, China). Multiwalled carbon nanotubes (MWCNT, diameter: 10-20 nm, length: 5-30 μm) and graphene oxide (GO, diameter: 0.5-5 μm, thickness: 0.8-1.2 nm) were purchased from XFNano Materials Technology Co., Ltd (Nanjing, China). Hydrochloric acid, cetyltrimethylammonium bromide (CTAB) and ammonia solution (25-28%) were purchased from Sinopharm Chemical Reagent Co., Ltd (Shanghai, China). Commercial solutions of superhydrophobic silica particles (SiO_2 NP) were obtained from Schanda Chemical Co., Ltd (Foshan, China). Phosphate buffer saline (PBS) was obtained from Sigma-Aldrich (USA). Primary human umbilical vein endothelial cell (HUVEC) and endothelial culture medium (ECM) were obtained from Scien Cell (USA). Hydrogen peroxide (H_2O_2), horseradish peroxidase (HRP), and Amplex Red were obtained from Beyotime Biotechnology Co. Ltd (Shanghai, China). All reagents were used as received without further purification. Ultrapure water with resistivity of 18.2 MΩ·cm was used in the studies provided by a Purelab flex water purification system (High Wycombe, UK).

1.2 Preparation of liquid metal particles

The liquid metal particles (LMPs) were prepared according to previous work.[1] Typically, 150 mg LM was added into 15 mL aqueous solution with 1 mg mL^{-1} methyl cellulose. Then, the mixture underwent sonication (KQ5200DE, 200 W) in ice-bath water for 1 hour. Afterwards, residual methyl cellulose in the dispersion was removed by centrifugation at 10,000 rpm for 10 min. Finally, the LMPs were dispersed in DMSO to obtain dark solutions, which were used in the following experiments.

1.3 Synthesis of gold nanorods

The gold nanorods (AuNRs) were synthesized according to our previous work.[2] Firstly, 0.1 M hydrochloric acid, 0.1 M CTAB, 0.4 mM chloroauric acid, 0.21 mM silver nitrate, and 5.26 mM hydroquinone were dissolved and maintained at 30 °C in an incubator. Then, 0.0017 mM sodium borohydride solution was slowly added to the above solution and then stirred for 8 h. AuNRs were obtained and then washed with water for three times. Finally, the AuNRs were dispersed in DMSO, which was used in the following experiments.

1.4 Preparation of silicon molds with inverse pyramidal arrays

The silicon mold is prepared according to our previous work.[3] As follows, 1-μm positive photoresist (AZ5214, Merck) film is formed via spin-coating (4000 rpm for 30 s) on a (100) silicon wafer with a 350-nm SiNx layer via sputtering deposition, followed by UV exposure (Karl Suss MA6) and developing processes. After RIE treatment for 5

min, the residual photoresist was removed by acetone. And the silicon wafer was then etched in potassium hydroxide (KOH) aqueous solution (80 °C) for 5 min for orientational erosion. After rinsed with distilled water and dried with N_2 flow, the silicon mold with inverse pyramidal arrays in its surface was obtained.

1.5 Preparation of P(VDF-TrFE) films and photothermal agent/P(VDF-TrFE) composite films

For P(VDF-TrFE) films, P(VDF-TrFE) powders were dissolved in DMSO at a concentration of 100 mg mL^{-1}, and the resulted solutions were further stirred at a rate of 300 rpm overnight. Then, the solutions were cast onto pre-cleaned pristine silicon wafers or silicon molds with inverse pyramidal arrays and dried at 80 °C for 12 h, and subsequently annealed at 130 °C for 8 h in a vacuum oven. Afterwards, the P(VDF-TrFE) films were released from the substrates after immersing in water, and then dried at room temperature (25 °C).[3]

For photothermal agent/P(VDF-TrFE) composite films, the LMPs, AuNRs, MWCNT and GO were dispersed into the P(VDF-TrFE) solutions (DMSO using as the solvent), respectively. Various composite films with LMPs ($m_{LMPs}/m_{P(VDF\text{-}TrFE)}$, from 1%, 2.5%, 5%, 10% to 15%), AuNRs (20 nM), MWCNT ($m_{MWCNT}/m_{P(VDF\text{-}TrFE)}$, 1%), and GO ($m_{GO}/m_{P(VDF\text{-}TrFE)}$, 0.5%) were prepared in the same manner mentioned above.

Finally, the films were poled by corona poling for 25 min at room temperature (25 °C).

1.6 Surface potential measurements

The scanning Kelvin probe microscopy (SKPM) is a universal tool for detecting regional surface potential variations across a substrate. The SKPM works by measuring the difference in work function between a movable probe and a fixed substrate. When the probe is moved close to the substrate surface, any difference in potential between the two leads them to exert a mutual force on one another. By modulating the applied potential on the probe until the mutual force is extinguished, it is thus possible to determine the local potential of the surface.

To evaluate the surface potential of the LMPs/P(VDF-TrFE) composite film, SKPM (Asylum Research Cypher S) measurements were performed under 808-nm near infrared (NIR) laser irradiation with various power densities of 4 mW cm^{-2}, 6 mW cm^{-2}, and 8 mW cm^{-2}, respectively. The initial surface potential of the composite film keeps approximating zero by partial ground connection to the surface which was coated with conductive silver paste. The scanning area was near the silver paste coating. The change of surface potential of the LMPs/P(VDF-TrFE) composite film can be recorded via periodical 808-nm NIR irradiation with interval time of 2.5 min at room temperature (25 °C). The surface potential is given by

$$U = \frac{Q}{C} \quad (1)$$

where Q is the surface charge generated by photo-induced pyroelectric effect, and C is the capacitance. Thus, the surface charge density of a given film increases as increasing the potential resulted from the increased power density of NIR light under a given irradiated area.

1.7 Electrical property measurement

In order to measure the photo-induced pyroelectric voltage and current, indium-tin oxide (ITO) electrodes were prepared via sputtering (100 W, 3 × 10^{-6} Torr, 1550 s; Lesker PVD 75) ITO on both sides of the P(VDF-TrFE) and photothermal agent/P(VDF-TrFE) composite films at room temperature, respectively. The temperature oscillations of the above films were performed by periodical irradiation with 808-nm NIR light using a controlled shutter, and the resulted open-circuit voltage and short-circuit current were recorded by a digital source meter (Keithley 2470, USA), respectively. Unless specifically mentioned, the electrical property of the above films was measured under constant relative humidity (~70%) and room temperature (25 °C). The temperature change was measured at the irradiated electrode surface using an infrared thermometer (FLIR E75, USA).

According to the pyroelectric effect, the photo-induced pyroelectric current is determined by following equation:

$$I = pS\frac{dT}{dt} \quad (2)$$

where p is the pyroelectric coefficient, S is the irradiated area, and $(\frac{dT}{dt})$ is the temperature change rates, respectively. During the time interval within the temperature change, the transferred charge can be derived as

$$Q = \int Idt = pS\Delta T \quad (3)$$

where Q is the transferred charge, and ΔT is the temperature change. According to Eq. (3), Q can be obtained by an integral of I. Thus, we can obtain charge density $(\frac{Q}{S})$ and current density $(\frac{I}{S})$ according to the above equations.

Additionally, the electrical property measurements under different conditions, including different relative humidity (30%, 50%, 70%, 90%), different environmental temperature (25 °C, 30 °C, 40 °C, 50 °C, 60 °C, 70 °C), different laser power density (28 mW cm^{-2}, 32 mW cm^{-2}, 42 mW cm^{-2}, 52 mW cm^{-2}, 76 mW cm^{-2}), and different irradiation angle (15°, 30°, 45°, 60°, 75°, 90°) were performed in the same manner mentioned above.

1.8 Light control of droplets

Firstly, the superamphiphobic LMPs/P(VDF-TrFE) films (pyramid and smooth films with poled or non-poled treatment) were obtained by further spraying commercial solutions of superhydrophobic SiO_2 particles on the surfaces. Then, the films were placed in a desiccator with 2 mL of TEOS and 2 mL of ammonia solution for 24 hours under vacuum condition. Finally, the films were further treated with air plasma (PDC-M, Suzhou Chemical Instrument Co., Ltd.) for 1 min, and afterwards deposited with 0.3 mL of PFDTS in vacuum for 24 hours to decrease the surface energy.[4,5]

For the light driven droplet motions, an 808-nm NIR laser (5 W, 6-mm spot diameter, Nanjing Laichuang Laser Technology Co., Ltd.) is used to manipulate the droplet on the previous superamphiphobic LMPs/P(VDF-TrFE) films (pyramid and smooth films with poled or non-poled treatment). The laser was fixed on a precise motion control platform (CL-01A, Haijie Technology Co., Ltd.) to control the laser moving velocities from 11.5 mm s^{-1}, 23 mm s^{-1}, 34.5 mm s^{-1}, 46 mm s^{-1} to 57.5 mm s^{-1}. The irradiation angle between the laser and the horizontal direction was tuned from 10°, 20°, 30°, 40°,

45°, 50°, 60°, 70°, 80° to 85°. Afterwards, various droplets (water droplets with various volumes from 1 μL, 3 μL, 5 μL, 8 μL, 10 μL, 20 μL to 40 μL; NaCl aqueous solutions with various concentrations from 0.1 M, 0.3 M, 0.5 M, 0.7 M to 1.0 M; glycerol aqueous solutions with various concentrations from 20 v/v%, 50 v/v% to 80 v/v%; EG and BD droplets) were dropped on the former superamphiphobic films, of which motions were performed under NIR irradiation at room temperature (25 °C) and recorded by a digital camera (Canon EOS 7D Mark II, Japan).

Typically, the 8-μL droplet motion cycles were carried out under the constant relative humidity (70%), room temperature (25 °C), laser moving velocity (34.5 mm s^{-1}), irradiation angle (45°) and vertical distance (3 cm) between the NIR laser and LMPs/P(VDF-TrFE) film. The movements of 8-μL droplet at different relative humidity (30%, 50%, 70%) were carried out under the same condition (irradiation angle, 45°; laser moving velocity, 34.5 mm s^{-1}; vertical distance between the NIR laser and film, 3 cm). For collective fusion, there dyed water droplets (4 μL) were manipulated by a handheld NIR laser pointer (FU808AD1000-GD22, SZ laser, 1000 mW, Shenzhen Zhonglai Technology Co., Ltd.). For the demonstrations, the water droplets (2 μL) colored by food dyes were manipulated by a handheld NIR laser pointer (ZLM50AD808-22130BXS, SZ laser, 50 mW, Shenzhen Zhonglai Technology Co., Ltd.) and recorded by a digital camera (Canon EOS 7D Mark II, Japan). The same driving conditions were also applied to droplets of $CaCl_2$ (1 M, 1 μL) and SA (2.0 wt%, 2 μL) aqueous solutions, which enables the formation of calcium cross-linked hydrogel beads with various morphologies.[6] The motions of droplets (2 μL) on the S-shaped

surface were recorded by a digital high-speed camera (Memrecam HX-7s, NAC, Japan) at a typical recording speed of 2,000 fps.

For the movement of a droplet with live cells in endothelial culture medium (ECM, ScienCell, USA), primary human umbilical vein endothelial cells (HUVECs) were used in the experiments. The cultured HUVECs were maintained in a 37 °C and 5% CO_2 incubator (Thermo Fisher Scientific, USA) with a humidified atmosphere. At passage 2 and 3 with 70% cell confluence, HUVECs were trypsinized and then suspended into an ECM with a cell density of 1×10^4 cells/mL. The HUVECs in the ECM were stained by using a LIVE/DEAD viability kit (Invitrogen, UK), where live and dead cells were labelled in green and red by calcein acetoxymethylester (Calcein-AM) ethidium homodimer-1 (EthD-1), respectively.[7,8] 2-μL ECM droplet with live cells was pipetted, and then dripped onto the film for light-driven motion. The handheld NIR laser pointer (ZLM50AD808-22130BXS, SZ laser, 50 mW, Shenzhen Zhonglai Technology Co., Ltd.) was used to drive the droplet to move forward on the film, which was recorded by a digital camera (EOS 7D Mark II, Canon, Japan). Before and after the motion of droplets driven by NIR irradiation treatments, the viability of the labelled HUVECs in the droplet was separately evaluated through the observation under a fluorescence microscope (Ni-U, Nikon, Japan).

To examine the biosensing performance, we firstly prepared a mixture solution of by diluting HRP and Amplex Red (a stock solution with a concentration of 10 mM dissolved in DMSO) with PBS into final concentrations of 0.2 U/mL and 100 μM, respectively (liquid A). Then H_2O_2 solutions (liquid B) of gradient concentrations (0,

6.25, 12.5, 25, and 50 μM) were prepared by diluting a stabilized H_2O_2 solution (1 M) with PBS and stored in ice bath. The droplets of liquid A (2 μL) and liquid B (2 μL) were separately pipetted and then guided for fusion by NIR laser beam (test). In control, the PICS film was pre-heated (ΔT: 60 K) for 30 s prior to the fusion of the two droplets. The infused droplets were observed using a fluorescence microscope after incubation at room temperature in dark for 30 min. The fluorescence intensities were measured using the ImageJ software from the fluorescence images obtained from three separate experiments. The curves demonstrating the fluorescence intensity with respective to the H_2O_2 solution concentration were then plotted.

1.9 Characterizations

The morphologies of the LMPs/P(VDF-TrFE) films were observed by a field emission scanning electron microscopy (SEM, Carl Zeiss Sigma 300, Germany). Energy Dispersive Spectrometer (EDS) elemental maps were obtained using a Bruker XFlash 6|60 detector. The 808-nm laser intensity was detected using an optical power meter (Thorlabs PM100D) equipped with a photodiode sensor (Thorlabs S425C; diameter, 25.4 mm; wavelength range, 190 nm-20 μm; power range, 2 mW-10 W). The optical images of LMPs/P(VDF-TrFE) films and droplet motions were taken by a digital camera (Canon EOS 7D Mark II, Japan). The infrared thermal imaging pictures together with the high-resolution pictures demonstrating the light-triggered thermogenesis of the LMPs/P(VDF-TrFE) films upon NIR irradiation were recorded using an infrared thermometer (R300SR, NEC, Japan), and an infrared thermometer

(R550Pro, 21 μm, NEC, Japan), respectively. To evaluate the surface morphologies and potentials of the LMPs/P(VDF-TrFE) composited film, Scanning Kelvin probe force microscopy (SKPM, Asylum Research Cypher S) was used in this experiment. The fluorescence images were taken by a fluorescence microscope (Ni-U, Nikon, Japan).

2. Discussion

2.1 Calculation of photothermal conversion efficiency

The total heat (Q) generated at the irradiated surface of the LMPs/P(VDF-TrFE) film is calculated as[9]

$$Q = cm\Delta T \tag{4}$$

where *c*, *m*, and ΔT are the specific heat (1.12 J g^{-1} K^{-1}), mass, and the temperature change.

The total energy output of the NIR laser (E) is calculated as

$$E = PSt \tag{5}$$

where *P* is the power density of the laser, *S* is the irradiated area, and *t* is the irradiation time.

Thus, the light-to-heat energy conversion efficiency (η) of LMPs/P(VDF-TrFE) film is calculated as

$$\eta = \frac{Q}{E} = \frac{cm\Delta T}{PSt} \tag{6}$$

According to the infrared thermal images (Figure S8), the light-to-heat energy conversion efficiency (η) of 5% LMPs/P(VDF-TrFE) film is the highest due to the largest temperature change (ΔT) value as shown in Figure S9. This is because further

increasing the LMPs mass ratio to 15% leads to the aggregation of LMPs in the LMPs/P(VDF-TrFE) film. Correspondingly, the sizes and morphologies of the LMPs in the LMPs/P(VDF-TrFE) film will change, thus leading to the decrease of the photothermal conversion efficiency of the LMPs/P(VDF-TrFE) films.

Notably, the light energy (E = 0.21 J, detected by an optical power meter) from the laser irradiation is mainly divided into three parts. One part of light energy is transferred into the thermal energy ($Q = cm\Delta T$, 0.196 J) of the composite film, and the light-to-heat energy conversion efficiency (η_1) is ~93.3%. The second part of light energy is first transferred into the thermal energy, and then transferred into electrical energy ($W = UIt$, 4.5 × 10^{-6} J), and the light-to-heat-to-electric energy conversion efficiency (η_2) is ~0.002%. The third part of light energy is consumed by the environment (*e.g.*, light reflection and transmission by the film, thermal diffusion to the air, $E_1=E-Q-W$, 0.014 J), and the consumption efficiency (η_3) is ~6.67%. These results indicate that light-to-heat-to-electric energy conversion efficiency can be improved by employment of photothermal agent with high photothermal and thermal-conductive effect, and avoidance of additional thermal-consumption layer, which all have been fully considered in the rational design of our photo-induced charged surfaces (PICS).

2.2 Numerical simulation of the droplet dynamics on PICS

To fully understand the droplet manipulation process, finite element analysis (COMSOL Multiphysics version 5.6) is implemented to simulate the droplet dynamics in the electric field of free charges induced by a gaussian irradiated spot on the PICS

film (100 μm). To simplify the analysis, the droplet is seen as a sphere. Thus, the distributions of temperature, surface charge density, electric field strength, electric potential can be expressed as:

$$\begin{cases} \sigma = p\Delta T \\ \mathbf{E} = -\nabla V \\ \nabla \cdot (\varepsilon_0 \varepsilon \mathbf{E}) = \sigma dS \\ \mathbf{n}(\mathbf{D}_1 - \mathbf{D}_2) = \sigma \end{cases} \tag{7}$$

where σ, p, ΔT, $\mathbf{E}$, V, dS, ε_0, ε, $\mathbf{n}$, and $\mathbf{D}$ are the surface charge density, pyroelectric coefficient of LMPs/P(VDF-TrFE), temperature change, electric field strength, electric potential, differential of area, vacuum permittivity and relative permittivity of LMPs/P(VDF-TrFE), surface normal vector and electric displacement, respectively (Table S1).

The nonuniform electric field induces a dielectrophoretic force (F_e) on a sphere droplet upon exposure to NIR irradiation. Noting that the droplet behaviors on PICS can be mainly modulated by F_e because of the negligible viscous energy dissipation (F_{res}) and Marangoni force (F_M), attributing to the excellent superamphiphobicity of PICS and negligible temperature gradient in the droplet during movements, respectively (Figure S4, Figures S19-21).

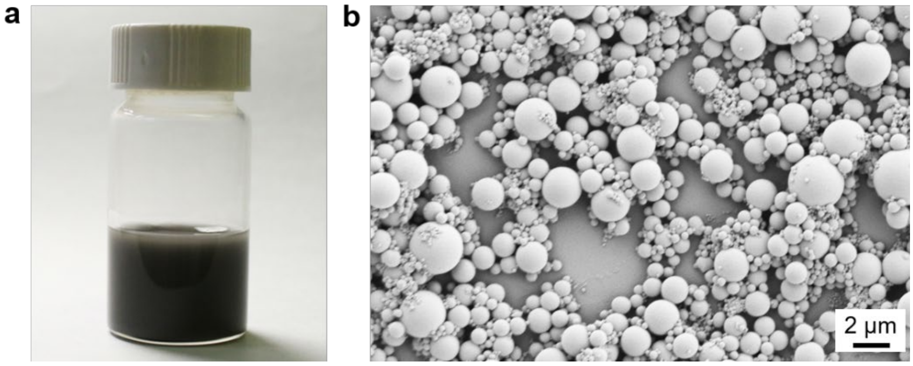

Figure S1. (a) Photographs of 5% LMPs/P(VDF-TrFE) suspension in DMSO. (b) SEM image of the synthesised LMPs.

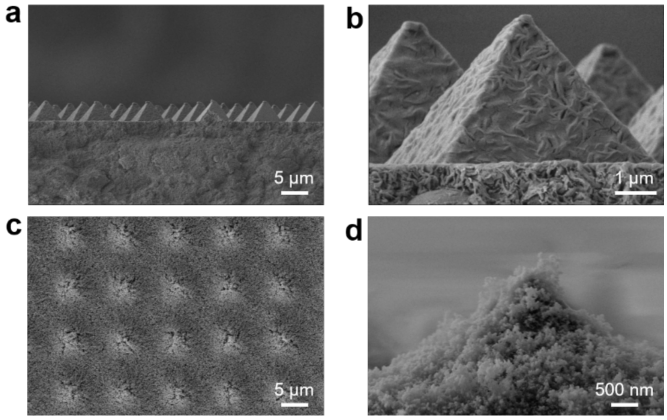

Figure S2. SEM images of the LMPs/P(VDF-TrFE) film. (a)-(b) Cross section of the film together with the enlarged image. (c)-(d) Top-down surface morphology of the film coated with fluorinated SiO_2 particles together with the side view of the enlarged image.

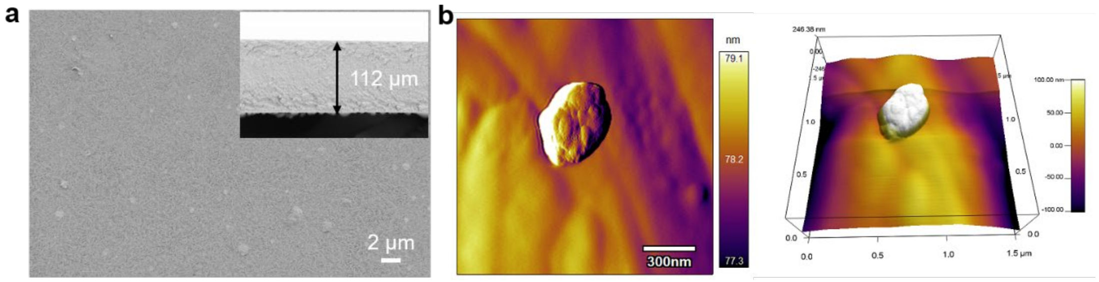

Figure S3. (a) SEM images of 5% LMPs/P(VDF-TrFE) film with thickness of 112 μm. (b) AFM images of 5% LMPs/P(VDF-TrFE) film.

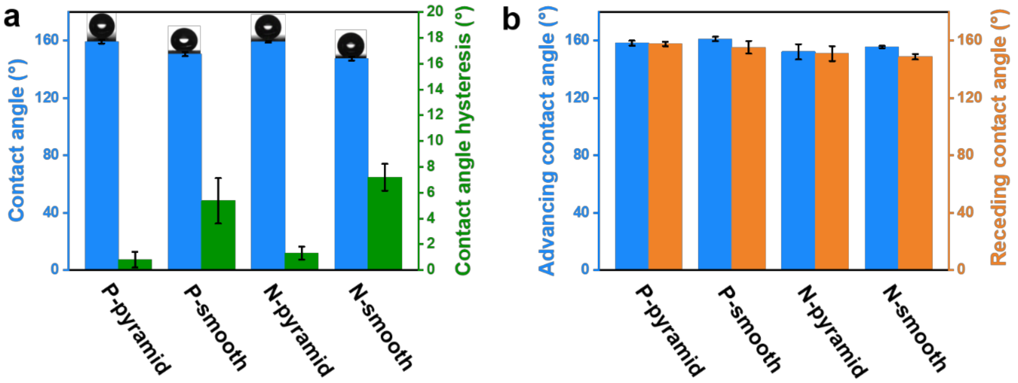

Figure S4. (a) The contact angles (CA) and contact angle hysteresis (CAH) of superamphiphobic LMPs/P(VDF-TrFE) films with poled pyramid surface (P-pyramid, CA: 159.4±1.3°, CAH: 0.8±0.6°), poled smooth surface (P-smooth, CA: 150.7±1.5°, CAH: 5.4±1.8°), non-poled pyramid surface (N-pyramid, CA: 159.2±0.5°, CAH: 1.3 ±0.5°), and non-poled smooth surface (N-smooth, CA: 147.6±1.4°, CAH: 7.2±1.0°). (b) The advancing contact angles (ACA) and receding contact angles (RCA) of superamphiphobic LMPs/P(VDF-TrFE) films with poled pyramid surface (P-pyramid, ACA: 158.3±1.7°, RCA: 157.8±1.5°), poled smooth surface (P-smooth, ACA: 161.2± 1.5°, RCA: 155.4±4.4°), non-poled pyramid surface (N-pyramid, ACA: 152.3±5.4°, RCA: 151.1±5.2°), and non-poled smooth surface (N-smooth, ACA: 155.7±0.8°, RCA: 148.8±1.9°).

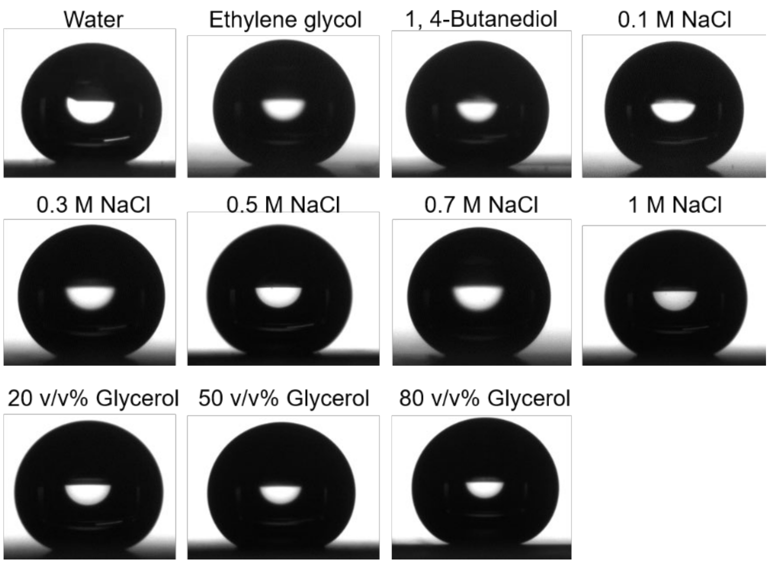

Figure S5. Static contact angles of various droplets (4 μL) on the superamphiphobic LMPs/P(VDF-TrFE) films (angle values listed in Table S2).

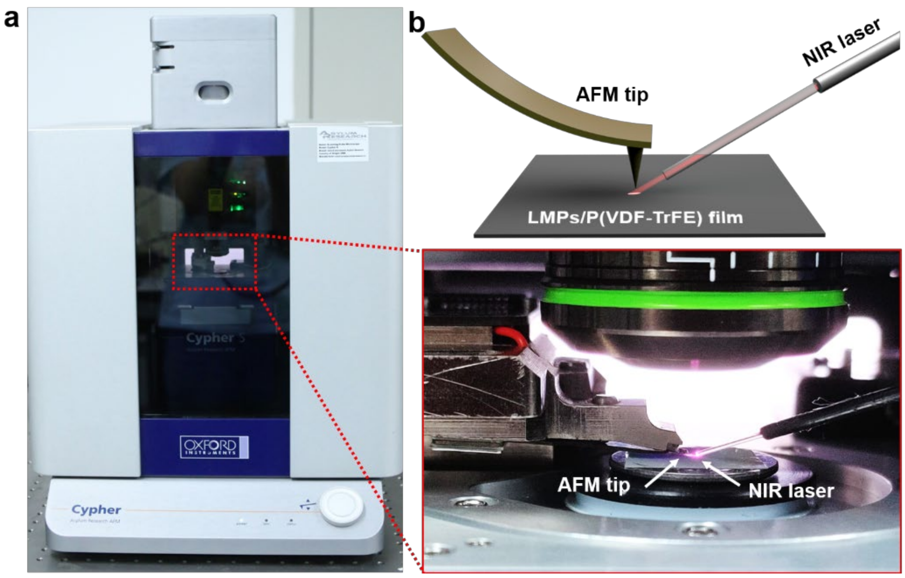

Figure S6. (a) The Kelvin probe force microscopy for characterizing the in-situ and real-time photo-induced charge generation of LMPs/P(VDF-TrFE) film. (b) Schematic illustration of photo-induced charge generation characterization.

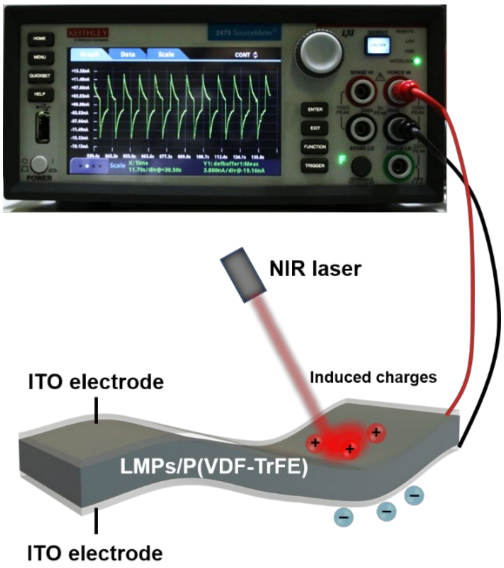

Figure S7. The setup for open-circuit voltage and close-circuit current characterization.

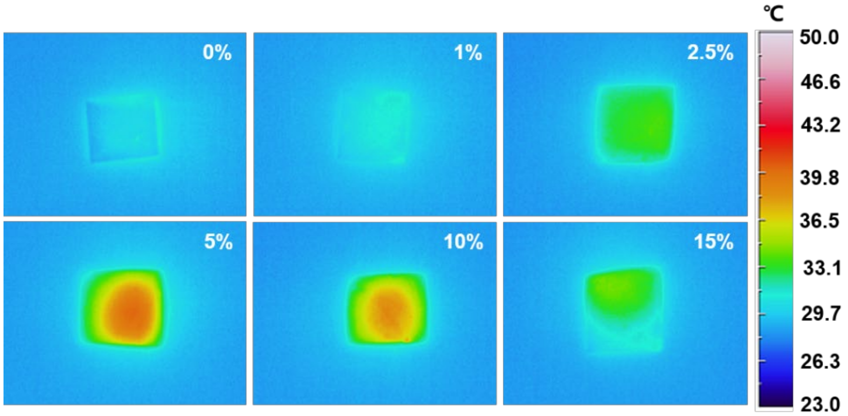

Figure S8. Infrared thermo imaging pictures of LMPs/P(VDF-TrFE) films with various concentrations of 0%, 1%, 2.5%, 5%, 10% and 15% as exposure to NIR irradiation with power density of 42 mW cm^{-2} for 5 s at room temperature. The 5% LMPs/P(VDF-TrFE) film shows the largest temperature change.

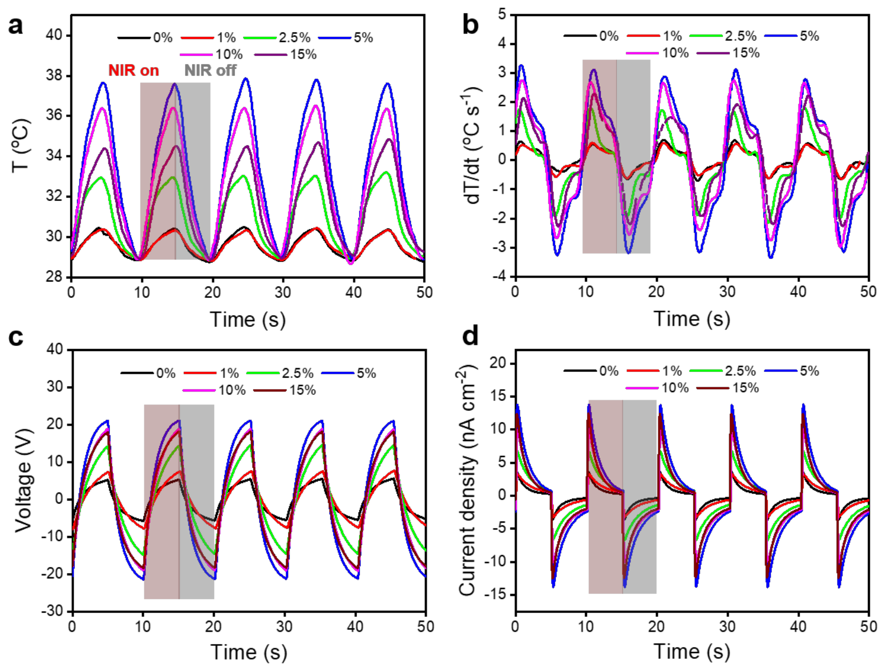

Figure S9. (a) The temperature change of LMPs/P(VDF-TrFE) films with various concentrations from 0% to 15% as exposure to NIR irradiation with power density of 42 mW cm^{-2} at temperature oscillation of 0.1 Hz at room temperature. (b) The temperature change rate of LMPs/P(VDF-TrFE) films with various concentrations from 0% to 15% as exposure to NIR irradiation with power density of 42 mW cm^{-2} at temperature oscillation of 0.1 Hz at room temperature. (c) The open-circuit voltage changes of LMPs/P(VDF-TrFE) films with various concentrations from 0% to 15% as exposure to NIR irradiation with power density of 42 mW cm^{-2} at temperature oscillation of 0.1 Hz at room temperature. (d) The closed-circuit current changes of LMPs/P(VDF-TrFE) films with various concentrations from 0% to 15% as exposure to NIR irradiation with power density of 42 mW cm^{-2} at temperature oscillation of 0.1 Hz at room temperature.

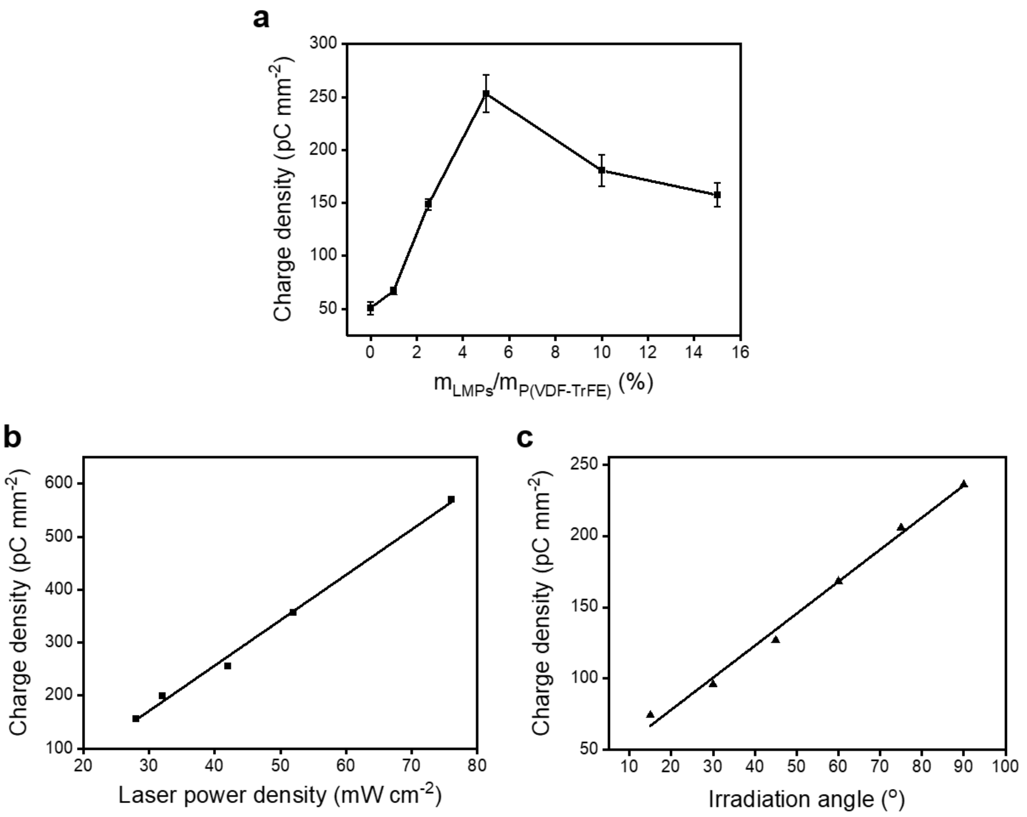

Figure S10. (a) The change of charge density from 50 pC mm^{-2} to 157 pC mm^{-2} with varying the concentrations from 0% to 15% as exposure to NIR irradiation with power density of 42 mW cm^{-2} for 5 s at room temperature. (b) The charge density increases from 156 pC mm^{-2} to 570 pC mm^{-2} with increasing the laser power densities from 28 mW cm^{-2} to 76 mW cm^{-2}. (c) The charge density increases from 74 pC mm^{-2} to 236 pC mm^{-2} with increasing the laser irradiation angle from 15° to 90° with a given irradiation distance between the laser and the LMPs/P(VDF-TrFE) film surface (12 cm).

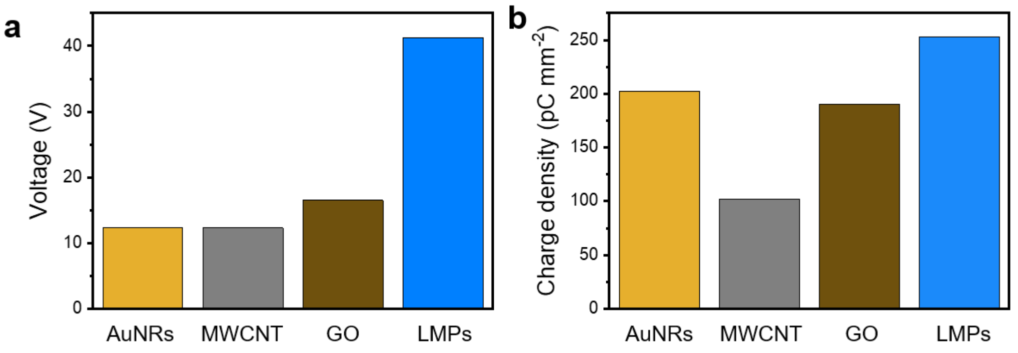

Figure S11. (a)-(b) Comparison of P(VDF-TrFE) films with various photothermal agents of 20 nM AuNRs, 1% MWCNT, 0.5% GO, and 5% LMPs upon exposure to NIR irradiation with power density of 42 mW cm^{-2} for 5 s at room temperature. The 5% LMPs/P(VDF-TrFE) film shows the largest open-circuit voltage and charge density.

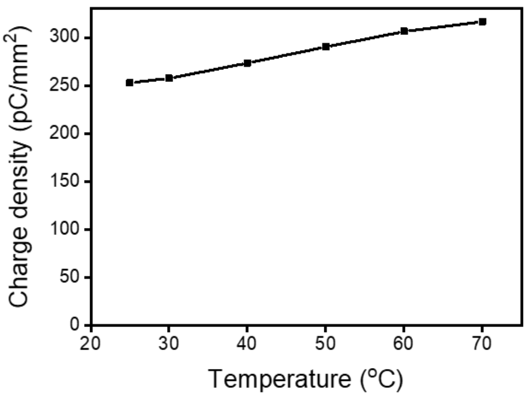

Figure S12. The charge density slightly changes from 253 pC mm^{-2} to 317 pC mm^{-2} with increasing the environmental temperature from 25 °C to 70 °C as exposure to NIR irradiation with power density of 42 mW cm^{-2} for 5 s.

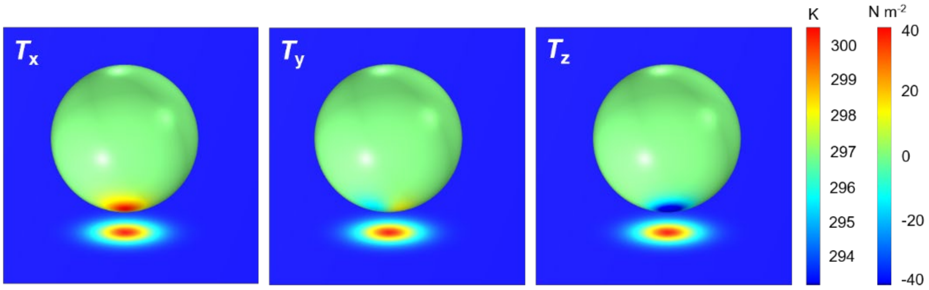

Figure S13. Maxwell stress tensors in x direction (T_x), y direction (T_y), and z direction (T_z) exerting on the droplet (8 μL) upon exposure to NIR irradiation (25 mW, irradiated spot radius: 0.4 mm, ΔT= 7 K) is obtained by COMSOL simulation.

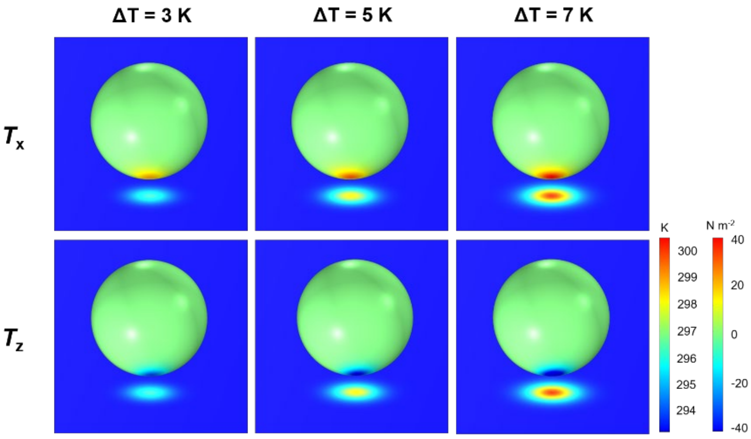

Figure S14. Maxwell stress tensors in x direction (T_x) and z direction (T_z) exerting on the droplet (8 μL) upon exposure to NIR irradiation (25 mW, irradiated spot radius: 0.4 mm), which increase via increasing the temperature change (ΔT, 3 K, 5 K, to 7 K).

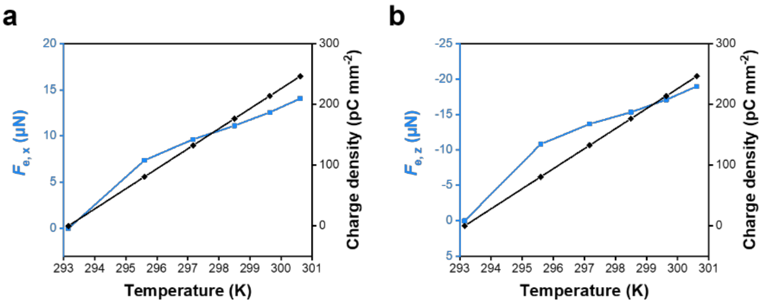

Figure S15. (a) The $F_{e,x}$, and $F_{e,z}$ integrated by the Maxwell stress tensors in x direction

(T_x) and z direction (T_z) respectively, can be adjusted by varying the temperature change (ΔT). (b) The charge density shows linear relationship with the temperature change.

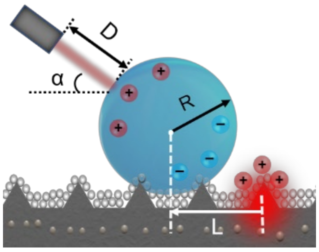

Figure S16. Schematic illustration of a droplet exposure to NIR irradiation. Varying the NIR irradiation angle (α) and the distance between the laser beam and the water droplet surface (D) leads to the change of the droplet position (L) and the photo-induced surface charge density.

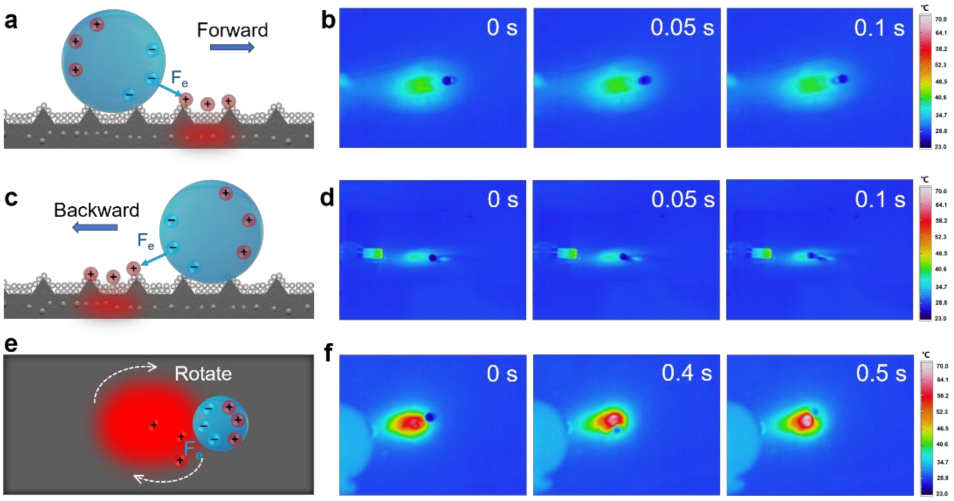

Figure S17. (a) Schematic illustration of a droplet moving forward as exposure to NIR irradiation. (b) Infrared thermo imaging pictures of an 8-μL droplet moving forward driven by NIR light at an irradiation angle of 45°. (c) Schematic illustration of a droplet moving backward as exposure to NIR irradiation. (d) Infrared thermo imaging pictures of an 8-μL droplet moving backward driven by NIR light at an irradiation angle of 15°.

(e) Schematic illustration of droplet rotation as exposure to NIR irradiation. (f) Infrared thermo imaging pictures of an 8-μL droplet rotation driven by NIR light at an irradiation angle of 80° (Movie S5).

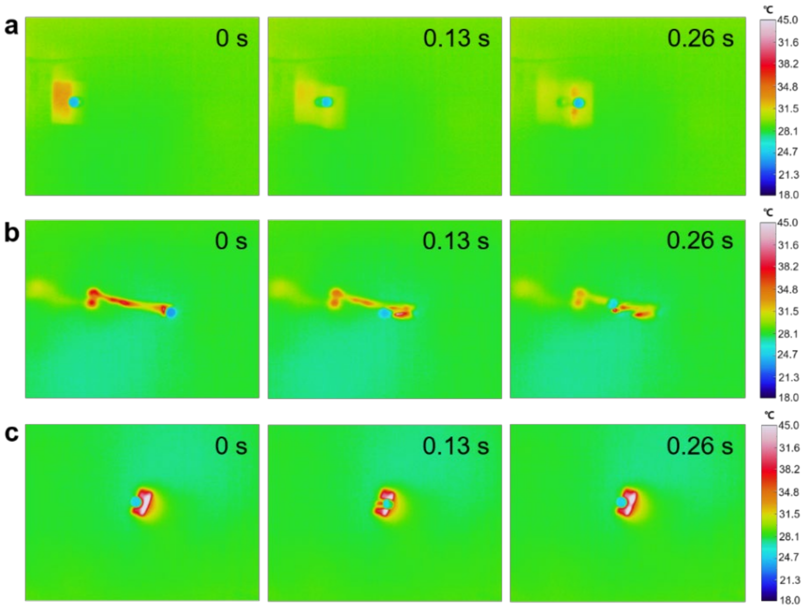

Figure S18. (a) Infrared thermo imaging pictures of a 4-μL droplet moving forward driven by a handheld laser pointer at an irradiation angle of 45°. (b) Infrared thermo imaging pictures of a 4-μL droplet moving backward driven by a handheld laser pointer at an irradiation angle of 15°. (c) Infrared thermo imaging pictures of a 4-μL droplet oscillating driven by a handheld laser pointer at an irradiation angle of 80° (Movie S6).

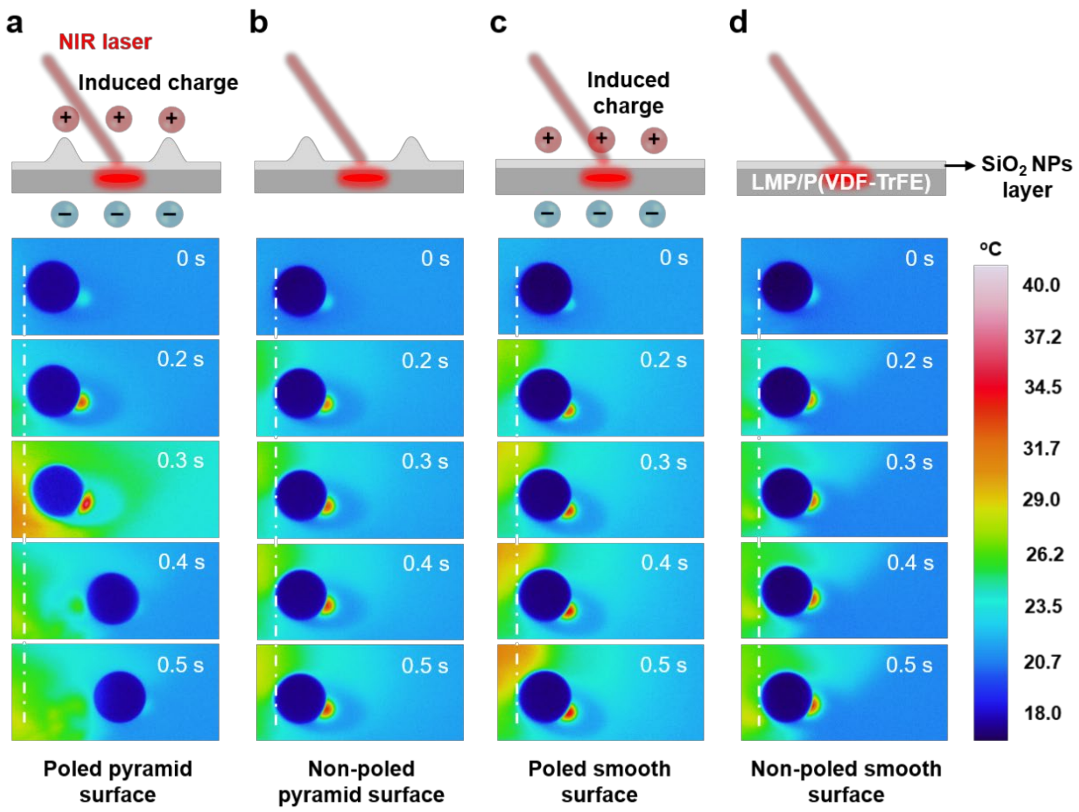

Figure S19. Infrared thermo imaging pictures of water droplets (8 μL) on various superamphiphobic LMPs/P(VDF-TrFE) films with (a) poled pyramid surface (P-pyramid), (b) non-poled pyramid surface (N-pyramid), (c) poled smooth surface (P-smooth), and (d) non-poled smooth surface (N-smooth), respectively. Although the temperature change is similar, the droplet cannot move on the non-poled pyramid surface, poled smooth surface and non-poled smooth surface as exposure to NIR irradiation (irradiation angle, 45°; power density, 996 mW cm^{-2}) for 0.5 s at room temperature due to the high resistance forces between the droplet and surfaces. Further increasing the power density of NIR irradiation, the droplet can move slowly on the poled smooth surface due to the increased dielectrophoretic force, however, the high interfacial resistance force still exist.

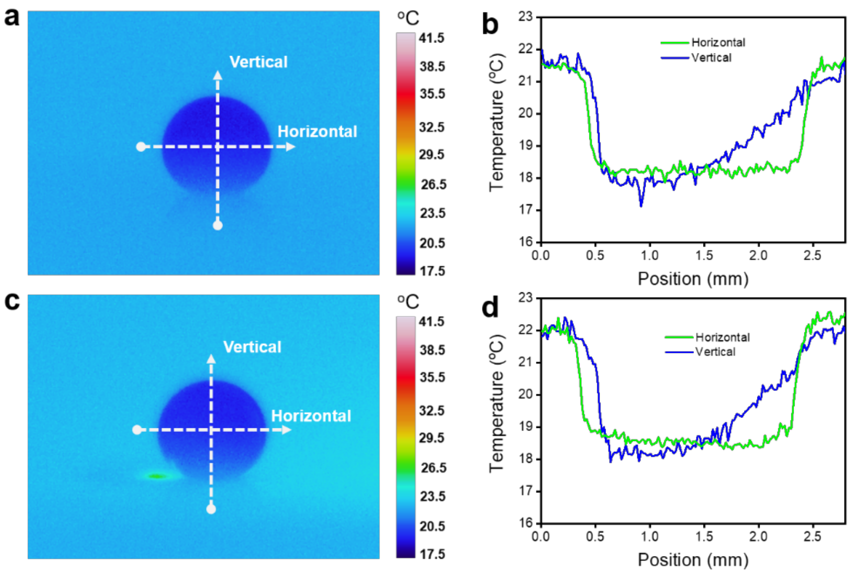

Figure S20. Infrared thermo imaging pictures and temperature distributions of two 8-μL water droplets on the superamphiphobic LMPs/P(VDF-TrFE) film without (a)-(b), and with (c)-(d) NIR irradiation at room temperature, respectively. And the temperature distribution of the moving water droplet under NIR irradiation (irradiation angle, 45°; average power density, 996 mW cm^{-2}) keeps consistent with the droplet without NIR irradiation, indicating that the photothermal effect has little effect on the temperature change of the water droplet due to the moving status.

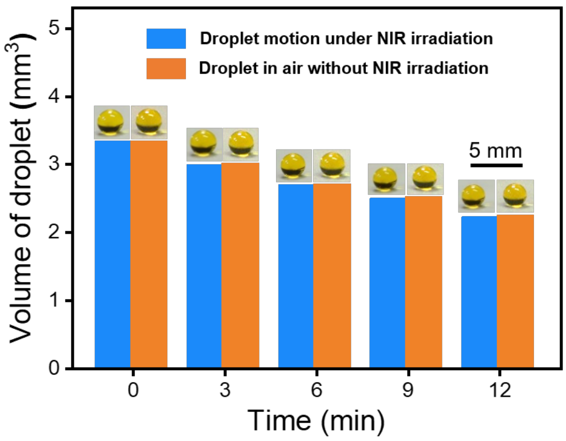

Figure S21. The volume changes of two 8-μL dyed water droplets on the superamphiphobic LMPs/P(VDF-TrFE) film with and without NIR irradiation for 12 minutes at room temperature, separately. The volumes of two droplets decrease as increasing the exposing time in air due to the evaporation of water. And the volume change of the moving water droplet under NIR irradiation (irradiation angle, 45°; average power density, 996 mW cm^{-2}) keeps consistent with the droplet without NIR irradiation, indicating that the photothermal effect has little effect on the water evaporation.

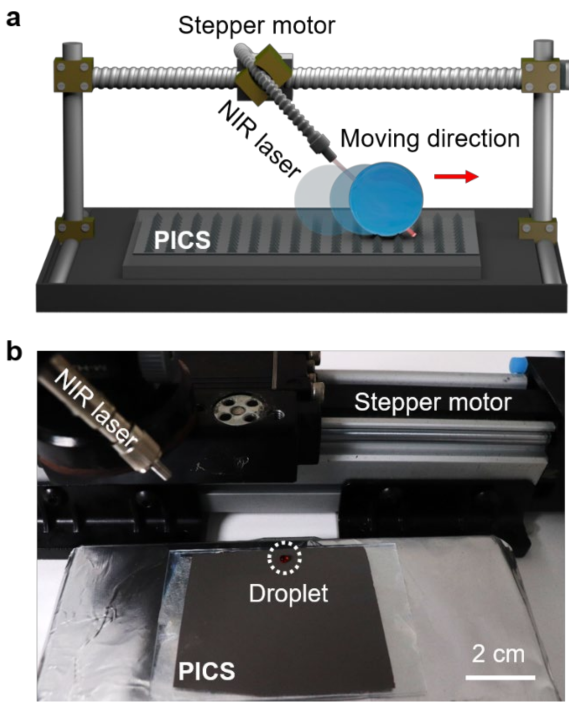

Figure S22. (a) Scheme of an automatic setup to administer precise movement of a laser beam for accurate experimental analysis. (b) Picture of the digital motion control platform for administering precise movement of a laser beam to manipulate droplets.

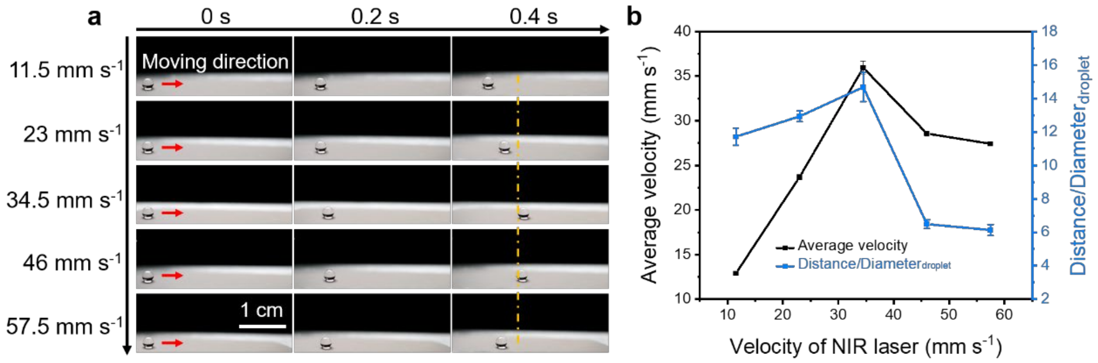

Figure S23. (a) Photographs of 8-μL droplet motion velocity changes via changing the laser moving speed, which was realised by the digital motion control platform. (b) The 8-μL droplet motion velocity changes from 12.9 mm s^{-1} to 27.4 mm s^{-1} with varying the laser moving speed from 11.5 mm s^{-1} to 57.5 mm s^{-1} as exposure to NIR irradiation (irradiation angle, 45°; power density, 996 mW cm^{-2}) at room temperature, thus

changing the motion distance to droplet diameter ratio correspondingly.

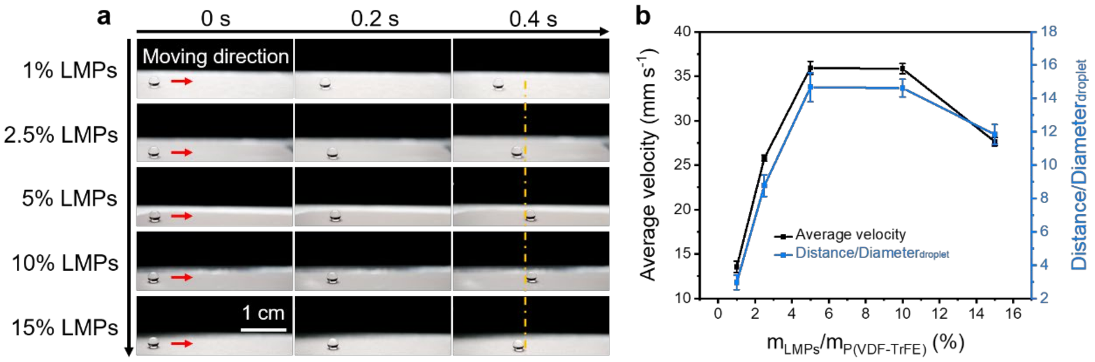

Figure S24. (a) Photographs of 8-μL droplet motions on LMPs/P(VDF-TrFE) films with various concentration of LMPs from 1% to 15%. (b) The 8-μL droplet motion velocity changes from 13.5 mm s^{-1} to 27.6 mm s^{-1} with varying the concentration of LMPs from 1% to 15% as exposure to NIR irradiation (irradiation angle, 45°; power density, 996 mW cm^{-2}) at room temperature, thus changing the motion distance to droplet diameter ratio correspondingly.

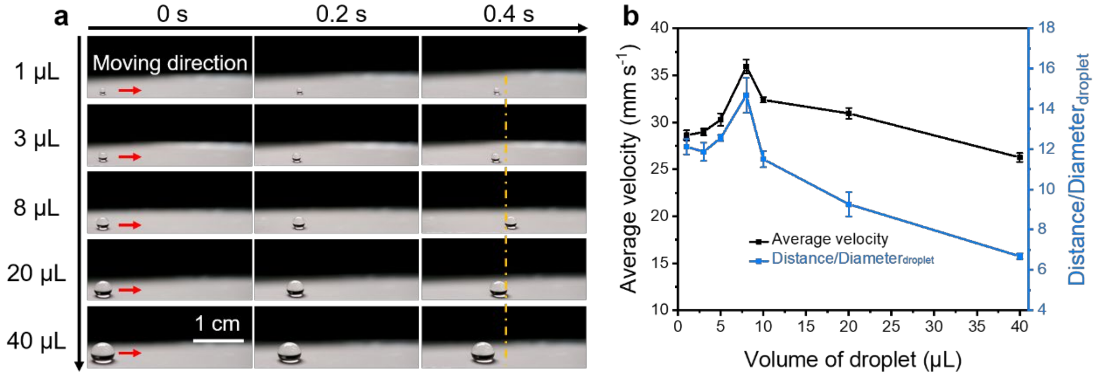

Figure S25. (a) Photographs of droplet motions with various droplet volumes from 1 μL to 40 μL on the 5% LMPs/P(VDF-TrFE) film. (b) The droplet motion velocity changes from 28.6 mm s^{-1} to 26.3 mm s^{-1} with varying the volume from 1 μL to 40 μL as exposure to NIR irradiation (irradiation angle, 45°; power density, 996 mW cm^{-2}) at

room temperature, thus changing the motion distance to droplet diameter ratio correspondingly.

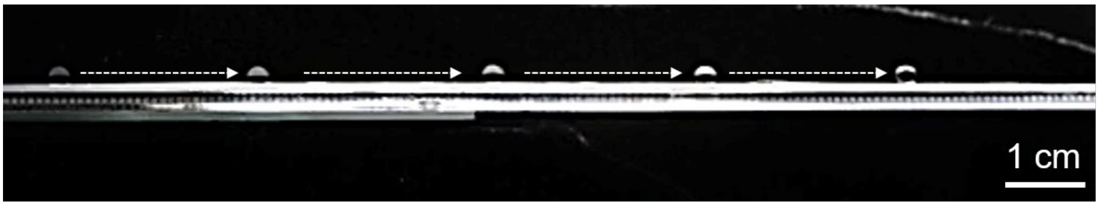

Figure S26. The time-lapse trajectory of long-distance motion of an 8-μL water droplet on PICS driven by 808-nm NIR laser, which was administered by the digital motion control platform (Movie S7).

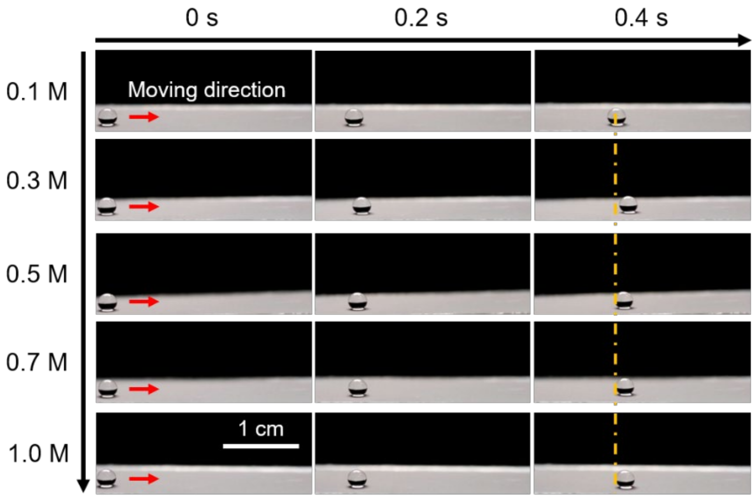

Figure S27. 8-μL droplets with various concentrations of NaCl from 0.1 M to 1.0 M move forward upon exposure to NIR light irradiation (irradiation angle, 45°; power density, 996 mW cm^{-2}) at room temperature, of which velocity changes from 33.3 mm s^{-1} to 34.3 mm s^{-1} correspondingly.

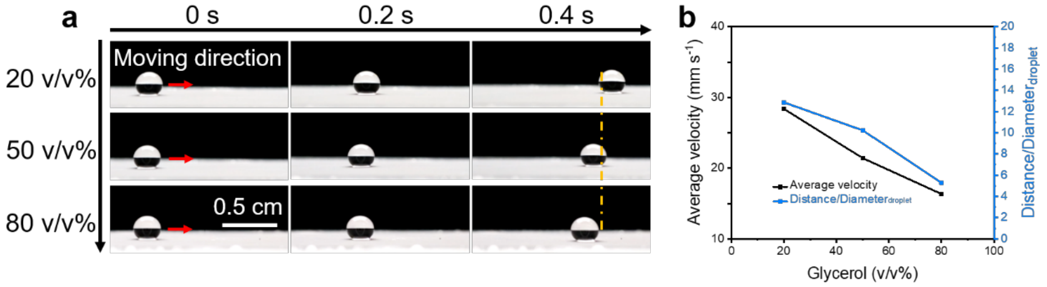

Figure S28. 8-μL droplets with various concentrations of glycerol from 20 v/v% to 80 v/v% move forward upon exposure to NIR light irradiation (irradiation angle, 45°; power density, 996 mW cm^{-2}) at room temperature, of which velocity changes from 28.4.3 mm s^{-1} to 16.3 mm s^{-1} correspondingly.

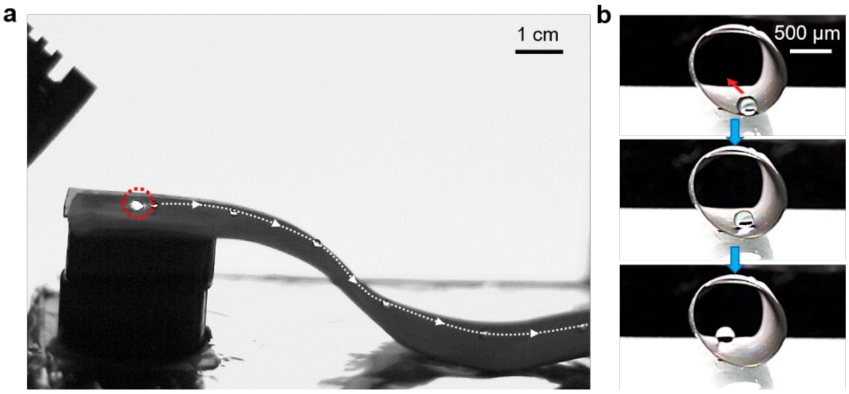

Figure S29. (a)-(b) Photographs of water droplets moving on S- (left) and O-shaped (right) films guided by a laser beam.

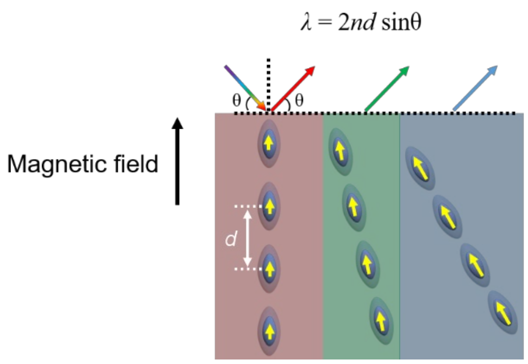

Figure S30. Schematic illustration of the color change mechanism of monodisperse Fe_2O_3@SiO_2 ellipsoidal core-shell particles as exposure to a magnetic field. The color (structural color) is resulted from the self-assembling of Fe_2O_3@SiO_2 ellipsoidal core-shell particles into periodical structures as exposure to a magnetic field. By changing the observation angle (θ) or the magnitude (or direction, thus changing the interparticle distance d) of the magnetic field, the Bragg diffraction color will change due to the changing λ according to the Bragg diffraction law.

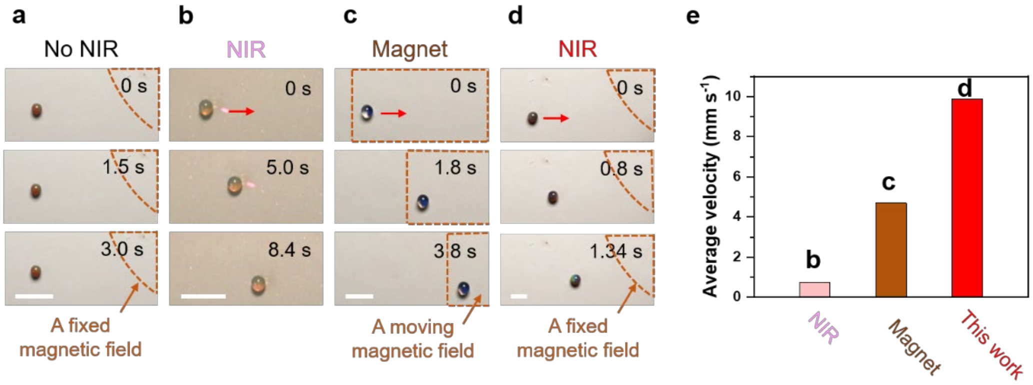

Figure S31. Performance of a 2-μL droplet containing monodisperse Fe_2O_3@SiO_2 ellipsoidal core-shell particles on PICS. (a) Without NIR light irradiation, the droplet cannot move even if there is a permanent magnet placed beneath the PICS film. (b) Upon exposure to NIR irradiation, the droplet can move slowly even when the permanent magnet is removed. (c) Without NIR light irradiation, the droplet can move

fast via firstly moving the permanent magnet near to the droplet, and then guiding the droplet. (d) Upon exposure to NIR light irradiation, the droplet can move very fast when the permanent magnet is placed beneath the PICS film. Scale bars: 5 mm. (e) Comparison of the average velocity of the droplet by the above manipulating conditions. These results indicate the droplet not only can be manipulated by a light-induced dielectrophoretic force via NIR light irradiation, but also can be guided by a large magnetic force via moving the permanent magnet nearby. Notably, the droplet possesses the maximum average velocity (~10 mm s^{-1}), attributing to the combined forces including the light-induced dielectrophoretic force and the magnetic force. On the contrary, the droplet remains stationary or moves slowly when there is no NIR irradiation or no magnetic field, respectively.

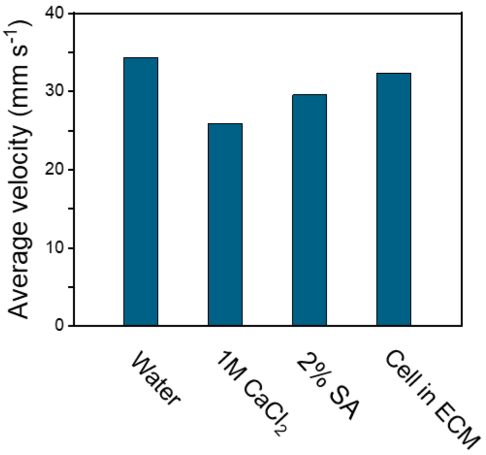

Figure S32. The average velocity of various droplets, including water (34.3 mm s^{-1}), 1 M $CaCl_2$ (25.9 mm s^{-1}), 2 wt% SA (29.6 mm s^{-1}), and cell suspension (32.4 mm s^{-1}).

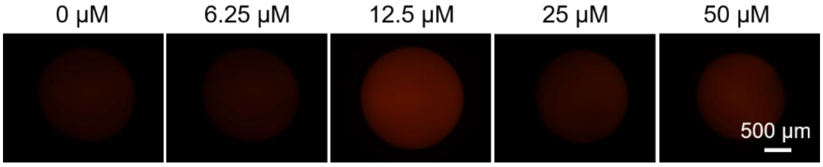

Figure S33. The fluorescence images of the control. The droplets contained H_2O_2 of different concentrations (0, 6.25, 12.5, 25, and 50 μM).

Table S1. Detail physical parameters used in the numerical simulation.

Notation	Expression	Description
T_0	293.15 K	Environment temperature
p	3.3×10^{-5} C/(K·m^2)	Pyroelectric coefficient
k	1.353 W/(m·K)	Thermal conductivity coefficient
e	0.9	Emissivity
ρ_w	1000 kg/m^3	Density of water
ε_w	81	Relative permittivity of water
ε_r	{7.4, 9.3, 7.6}	Relative permittivity of LMPs/P(VDF-TrFE)
ρ_p	1780 kg/m^3	Density of LMPs/P(VDF-TrFE)
C_p	1170 J/(kg·K)	Heat capacity at constant pressure
σ	0.4 mm	Radius of Gaussian laser beam
P_l	25 mW	Power of Gaussian laser beam

Table S2. Detailed parameters (different α and D) for manipulating droplets. L can be approximately calculated according to α, D, and droplet diameter. When $\alpha < 10°$, the L cannot be calculated owing to the divergence irradiation of NIR light.

α (°)	***D*** **(cm)**	***L*** **(mm)**	**Laser power density ($mW\ cm^{-2}$)**
85	4.35	0.11	683
80	4.45	0.22	648
70	5.10	0.45	626
60	5.85	0.71	549
50	6.85	1.03	500
45	7.21	1.23	462
40	6.43	1.47	481
30	6.24	2.13	618
20	5.99	3.38	866
10	4.24	-	503

Table S3. The motion speeds, static contact angle and contact angle hysteresis of various liquids on the superamphiphobic LMPs/P(VDF-TrFE) film together with their physical properties. Thus, the higher dielectric constant and surface tension the liquid has, the faster the droplet can be moved.[10,11]

Liquids	Velocity (mm s^{-1})	Volume (μL)	Dielectric constant (ε_r)	Density (kg m^{-3})	Surface tension (mN m^{-1})	Viscosity (mPa s)	Contact angle (°)	Contact angle hysteresis (°)
Water	35.9	8	81	1000	72.1	1	159.4 ± 1.3°	0.81± 0.6°
Ethylene glycol	10.4	2	37.7	1116	48.2	21.4	156.2 ± 2.4°	1.2 ± 0.9°
1,4-Butanediol	2.2	2	31.1	1017	39.6	88.8	153.5± 2.0°	3.3 ± 1.1°
0.1 M NaCl	33.3	8	78.7	1005	72.2	1	158.2±0.7	0.9±0.8
0.3 M NaCl	32.3	8	74.6	1012	72.5	1	158.0±2.3	1.0±0.9
0.5 M NaCl	34.3	8	73.4	1019.5	73.1	1	157.1±0.8	0.9±0.8
0.7 M NaCl	32.4	8	72	1023	73.4	1.1	157.8±1.4	1.0±1.0
1 M NaCl	34.3	8	95	1041	73.9	1.1	157.0±1.2	1.0±0.9
20 v/v% Glycerol	28.4	8	72.9	1047	71.7	1.76	157.4±1.1	0.9±0.9
50 v/v% Glycerol	21.4	8	62.3	1120	69.3	10.8	156.0±2.1	1.9±1.1
80 v/v% Glycerol	16.3	8	45.6	1190	66.5	60.1	155.13±0.6	2.6±1.3

Table S4. Comparison of the droplet motion performances among our photo-induced dielectrophoretic force strategy with the reported photomechanics, photochemistry, photothermal, heat, and light-induced electric field strategies.

Ref. in main text	Mechanism	Liquid	Maximum velocity (mm s^{-1})	Surface tension (mN m^{-1})	Viscosity (mPa s)	Volume of liquid (μL)	Laser wavelength (nm)
Ref. 12	Photomechanics	Hexane	5.9	18.4	0.3	0.3	470
Ref. 10	Photochemistry	Olive oil	0.03	33.1	12	2	365/436
Ref. 11	Photochemistry	Oleic acid	0.3	33.8	38.8	3	365/475
Ref. 13	Photothermal	Propylene glycol	1.7	38	56	2	808
Ref. 33	Photothermal	Liquid paraffin	8	0.024	2.5	10	532
Ref. 34	Heat	25 v/v% Propylene glycol	2	/	/	0.5	/
Ref. 35	Heat	Water	6.5	72.7	1	10	/
Ref. 36	Dielectrophoretic	Water	18.5	72.7	1	4	808
Ref. 5	Dielectrophoretic	Water	1	72.7	1	4	785
Ref. 14	Optoelectrowetting	Water	7	72.7	1	4	/
**Our work**	**PICS**	**Water**	**35.9**	**72.7**	**1**	**8**	**808**

3. Movies

Movie S1. Light control of water droplet fast motion with a high average velocity.

Movie S2. Light control of three dyed water droplets for collective motions.

Movie S3. Light control of a water droplet for rolling.

Movie S4. Light control of a water droplet for multimode motions.

Movie S5. Light control of a water droplet for forward, backward, and rotation motions (Thermal imaging video).

Movie S6. Light control of a water droplet for forward, backward, and oscillation motions (Thermal imaging video).

Movie S7. Light control of water droplet motion for ultralong distance.

Movie S8. Light control of ethylene glycol and 1,4-butanediol droplets.

Movie S9. Light control of a dyed water droplet for transporting a solid cargo in a closed tube.

Movie S10. Light control of a dyed water droplet for crossing a small-size tunnel.

Movie S11. Light control of a dyed water droplet for cleaning a powder sample.

Movie S12. Light control of a dyed water droplet for precise obstacle avoidance.

Movie S13. Light control of a color-changing droplet robot.

Movie S14. Light control of two droplets for merging into a snowman hydrogel bead.

4. References

1. Li, X. et al. Evaporation-induced sintering of liquid metal droplets with biological nanofibrils for flexible conductivity and responsive actuation. *Nat. Commun.* **10**, 1-9 (2019).
2. Du, X. et al. Photothermally Triggered shape-adaptable 3D flexible electronics. *Adv. Mater. Technol.* **2**, 1700120 (2017).
3. Wang, F. *et al.* Light-induced charged slippery surfaces. *Sci. Adv*. **8**, eabp9369 (2022).
4. Deng, X. et al. Candle soot as a template for a transparent robust superamphiphobic coating. *Science* **335**, 67-70 (2012).
5. Wang, J. et al. Tunable shape memory polymer mold for multiple microarray replications. *J. Mater. Chem. A* **6**, 24748-24755 (2018).
6. Du, X. et al. Inside-out 3D reversible ion-triggered shape-morphing hydrogels. *Research* **2019**, 6398296 (2019).
7. Zhao, Q., Wang, J., Wang, Y., Cui, H. & Du, X. A stage-specific cell manipulation platform for on-demand inducing endothelialization. *Natl. Sci. Rev.* **7**, 629-643 (2020).

8. Zhao, Q. et al. Programmed shape-morphing scaffolds enabling facile 3D endothelialization. *Adv. Funct. Mater.* **28**, 1801027 (2018).
9. Li, J. et al. Micropatterning of the Ferroelectric phase in a poly(vinylidene difluoride) film by plasmonic heating with gold nanocages. *Angew. Chem. Int. Ed.* **55**, 13828-13832 (2016).
10. Sun, Q. et al. Surface charge printing for programmed droplet transport. *Nat. Mater.* **18**, 936-941 (2019).
11. Li, W., et al., Photopyroelectric microfluidics. *Sci. Adv*. **6**, eabc1693 (2020).
